# Mitogen-Activated Protein Kinase Cascade MKK7-MPK6 Plays Important Roles in Plant Development and Regulates Shoot Branching by Phosphorylating PIN1 in *Arabidopsis*

**DOI:** 10.1371/journal.pbio.1002550

**Published:** 2016-09-12

**Authors:** Weiyan Jia, Baohua Li, Shujia Li, Yan Liang, Xiaowei Wu, Mei Ma, Jiyao Wang, Jin Gao, Yueyue Cai, Yuanya Zhang, Yingchun Wang, Jiayang Li, Yonghong Wang

**Affiliations:** 1 State Key Laboratory of Plant Genomics and National Center for Plant Gene Research, CAS Center for Excellence in Molecular Plant Sciences, Institute of Genetics and Developmental Biology, Chinese Academy of Sciences, Beijing, China; 2 State Key Laboratory of Molecular Developmental Biology, Institute of Genetics and Developmental Biology, Chinese Academy of Sciences, Beijing, China; UCSD, UNITED STATES

## Abstract

Emerging evidences exhibit that mitogen-activated protein kinase (MAPK/MPK) signaling pathways are connected with many aspects of plant development. The complexity of MAPK cascades raises challenges not only to identify the MAPK module *in planta* but also to define the specific role of an individual module. So far, our knowledge of MAPK signaling has been largely restricted to a small subset of MAPK cascades. Our previous study has characterized an *Arabidopsis bushy* and *dwarf1* (*bud1*) mutant, in which the MAP Kinase Kinase 7 (MKK7) was constitutively activated, resulting in multiple phenotypic alterations. In this study, we found that MPK3 and MPK6 are the substrates for phosphorylation by MKK7 *in planta*. Genetic analysis showed that MKK7-MPK6 cascade is specifically responsible for the regulation of shoot branching, hypocotyl gravitropism, filament elongation, and lateral root formation, while MKK7-MPK3 cascade is mainly involved in leaf morphology. We further demonstrated that the MKK7-MPK6 cascade controls shoot branching by phosphorylating Ser 337 on PIN1, which affects the basal localization of PIN1 in xylem parenchyma cells and polar auxin transport in the primary stem. Our results not only specify the functions of the MKK7-MPK6 cascade but also reveal a novel mechanism for PIN1 phosphorylation, establishing a molecular link between the MAPK cascade and auxin-regulated plant development.

## Abstract 2

The MKK7-MPK6 cascade plays a specific role in regulating auxin-related developmental events and phosphorylates PIN1, affecting its polarization and determining shoot branching in *Arabidopsis*.

## Author Summary

MAPK cascades play important roles in transducing environmental and developmental signals into adaptive and programmed responses. Because of the complexity of MAPK cascades, revealing the specificity of the MAPK modules is key to forming a functional and fully connected signal transduction system in higher plants. In the MAPK signaling module, MAPK kinases (MKKs) are of particular importance because they serve as the convergence and divergence points in the MAPK signal transduction. Our previous study had characterized an *Arabidopsis bushy* and *dwarf1* (*bud1*) mutant, in which the MAP Kinase Kinase 7 (MKK7) was constitutively activated, leading to multiple auxin-related developmental defects. Here, we used the *bud1* mutant to discover the signaling events of MKK7 downstream modules. Our results demonstrated that MPK6 and MPK3 are two major downstream targets of MKK7. Furthermore, we found that MKK7-MPK6 cascade phosphorylates the Ser 337 (S337) site of PIN1, affecting PIN1’s polar localization and thus modifying shoot branching. Our findings specify the functions of the MKK7-MPK6 cascade and explain how the MKK7-MPK6 signaling pathway regulates polar auxin transport to determine shoot branching in *Arabidopsis*.

## Introduction

Mitogen-activated protein kinase (MAPK/MPK) cascades play important roles in a broad spectrum of signals, including biotic and abiotic stresses and hormone-mediated development in higher plants [1]. The basic MAPK module is composed of three sequentially activated kinases: MAPK kinase kinase (MAPKKK), MAPK kinase (MKK), and MPK. MAPK could phosphorylate the downstream substrates to elicit biological responses to various developmental requirements and environmental stimuli [1]. The *Arabidopsis* genome encodes a large number of MAPK cascade components with more than 60 MAPKKKs, 10 MKKs, and 20 MPKs, which participate in regulating many essential biological processes [1,2].

In the MAPK signaling module, MKKs are of particular importance because they serve as the convergence and divergence points in the MAPK signal transduction [3,4]. Based on the protein microarray data, an overview of interactions between the MKKs and MPKs as well as between MPKs and the downstream substrates has been proposed [5,6], showing that an individual MKK could target multiple MPKs, and an individual MPK could be a substrate of multiple MKKs. Moreover, downstream substrates of MAPK cascades also determine the function of MAPK signaling. The complexity of MAPK cascades raises challenges not only to identify the MAPK module *in planta* but also to define the specific role of an individual module. As there are 10 MKKs and 20 MPKs in the *Arabidopsis* genome, the signaling specificity of the MAPK modules should partially rely on the diversity of the MPKs and their downstream signaling events. MPK3 and MPK6, the most intensively studied MPKs in *Arabidopsis*, have overlapping functions in diverse development and stress-related adaptation processes [7–16]. Although the different roles of MPK3 and MPK6 in certain biological events have been recently reported [17–23], the signaling specificity of the two MPKs in more diverse biological processes remains to be elucidated.

Plant growth regulator auxin is synthesized in the shoot apical and flows down through the vasculature of the primary stem to mediate plant development [24]. A subset of the *Arabidopsis* PIN-FORMED proteins (PINs) with long hydrophilic loop (HL), namely PIN1–PIN4, PIN6, and PIN7, localizes predominantly to the plasma membrane in diverse tissues and displays distinct subcellular polarity depending on PIN species and tissue types, determining the direction of auxin flow [24–26]. Among these PIN proteins, PIN1 is the major member that regulates shoot development in *Arabidopsis* [27]. Recent studies suggested that the PIN1 polar localization is related to its phosphorylation status [28–32]. The Ser/Thr (S/T) protein kinase PINOID (PID) and protein phosphatase 2A (PP2A) have been reported to mediate PIN1 apical-basal polarity by regulating PIN1 phosphorylation in an antagonistic manner [31,33]. The residues S231, S252, and S290 of PIN1 are directly phosphorylated by PID [29]. Although S337 and T340 were also shown to be essential for both PIN1 polar localization and auxin flow, they are not substrates of PID, implying that there might exist other protein kinases involved in PIN1 phosphorylation [32].

Our previous studies isolated a semidominant *bushy* and *dwarf 1* (*bud1*) mutant in *Arabidopsis*, which results from the overexpression of *MAP Kinase Kinase 7* (*MKK7*) [34]. The constitutively increased expression of MKK7 leads to multiple phenotypic changes, including enhanced gravitropism of dark-grown seedlings, fewer lateral roots, abnormal filament elongation, more branches, dwarfism, and smaller and curled leaves [34]. These diverse phenotypes in the *bud1* mutant imply that multiple MAPK signaling pathways may be activated by MKK7. Here, we demonstrated that MPK3 and MPK6 were two major downstream substrates of MKK7 in vitro and in vivo. Genetic analysis showed that MKK7-MPK6 and MKK7-MPK3 signaling pathways play distinct roles in plant development. The MKK7-MPK6 signaling pathway specifically regulates shoot branching, plant height, lateral roots development, flower filament elongation, hypocotyl gravitropism, and basipetal polar auxin transport in seedlings and main roots, whereas the MKK7-MPK3 signaling pathway specifically regulates leaf development. We further showed that PIN1 is the substrate of the MKK7-MPK6 cascade for phosphorylating at S337, which determines PIN1 polarization and regulates shoot branching in *Arabidopsis*.

## Results

### MKK7 Can Activate MPK3 and MPK6 In Vitro and In Vivo

**Fig 1:**
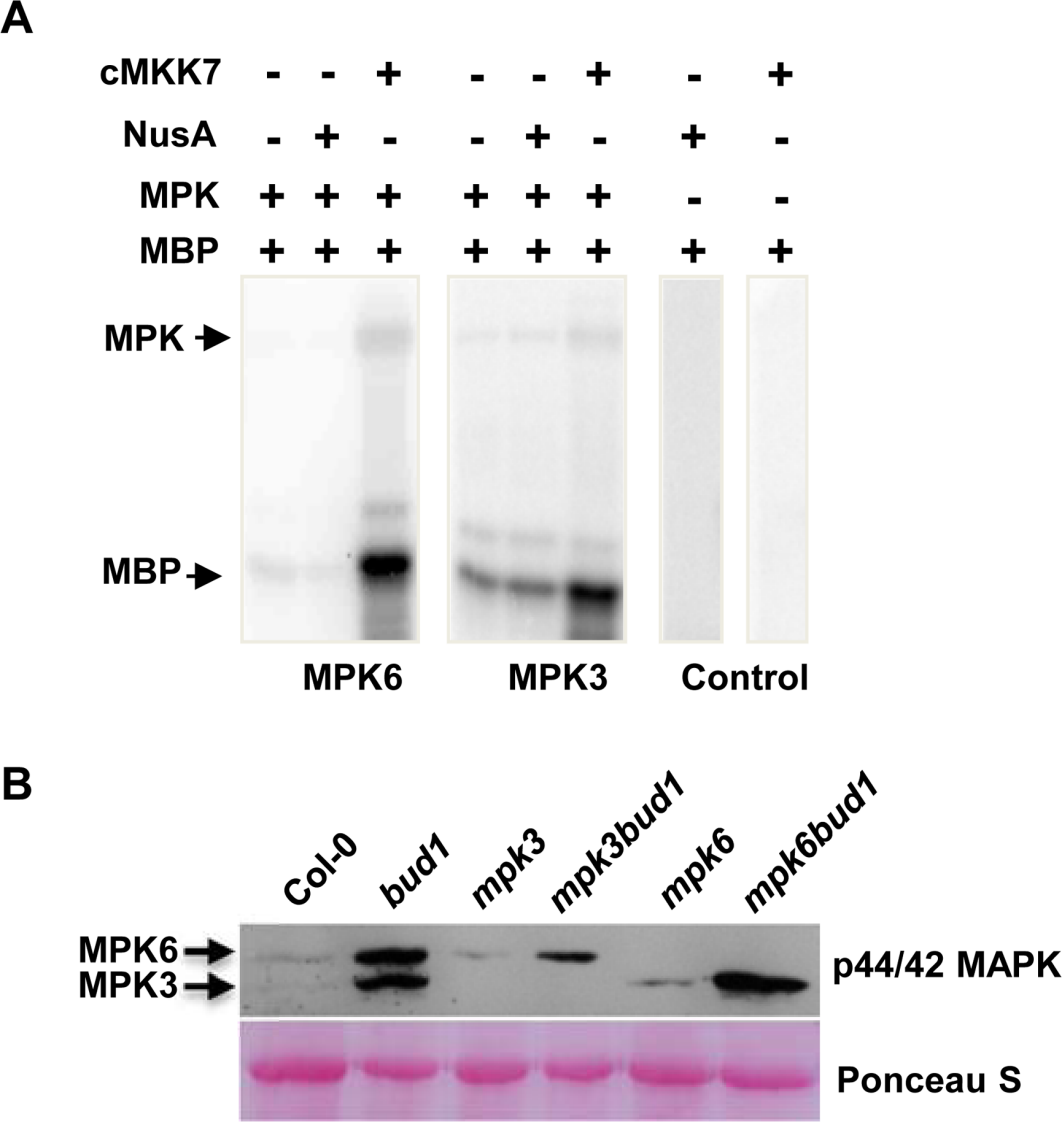
MKK7 can phosphorylate MPK3 and MPK6 in vitro and in vivo. (A) In vitro kinase assays of phosphorylation of MPK3 and MPK6 by constitutively activated MKK7 (cMKK7). The cMKK7 was incubated with MPK3 or MPK6 in the kinase reaction buffer. Aliquots of the samples were separated by SDS-PAGE and subjected to autoradiography. Arrows indicate positions of the detected proteins. (B) Phosphorylation of MPK3 and MPK6 *in planta*. Samples were prepared from 21-d-old seedlings and subjected to immunoblot analysis with antiphospho-p44/p42 antibody. The Ponceau-stained western blot was used as the loading control.

Although MKK7 was predicted to have the ability to activate multiple MPKs by different screening systems [6,35], there is no in vivo experimental evidence showing the relationships between MKK7 and its downstream MPKs. Using myelin basic protein (MBP) as the substrate of MPKs, we found that constitutively activated MKK7 (cMKK7) could activate MPK3 and MPK6 under tested experimental conditions (Fig 1A and S1 Fig), which is consistent with the previous report by Yoo et al. [9].

The activated MAPK system is a common approach to characterize downstream targets of MAPK cascade [13,36–38]. To verify that the phenotypes in *bud1* are not ectopic effects of *MKK7* overexpression, we identified a *MKK7* knockout mutant (S2A Fig). Compared with the wild type, the *mkk7* mutant exhibited significantly reduced shoot branch number, increased plant height and lateral root number (S2B–S2D Fig), enhanced polar auxin transport in inflorescence stems (S2E Fig), and longer hypocotyls at high temperature under light (S2F and S2G Fig). All of these phenotypes are opposite to those observed in *bud1*, which further supports our conclusion that MKK7 plays an important role in the regulation of shoot branching and other auxin-related developmental events, indicating that *bud1* can be used for characterizing MKK7 downstream targets.

To confirm the activation of MPK3 and MPK6 by MKK7 *in planta*, we analyzed the activation of MPKs in the *bud1* mutant using the phospho-p44/p42 antibody, which specifically recognizes the phosphorylated MPK3 and MPK6 [39]. The result showed that both MPK3 and MPK6 were phosphorylated in *bud1*, indicating that MKK7 could activate MPK3 and MPK6 in vivo (Fig 1B).

### Generation and Characterization of *mpk3bud1* and *mpk6bud1* Double Mutants

MPK3 and MPK6 belong to Group A MPKs [2] and share 68.69% sequence identity at the amino acid level (S3 Fig). To dissect the specific roles of MPK3 and MPK6 mediated by MKK7, we generated the double mutants of *mpk3bud1* and *mpk6bud1* by crossing the *bud1* mutant with the *mpk3* or *mpk6* single mutant, respectively. The *mpk3* mutant is a deletion mutant caused by fast neutron mutagenesis [40], and the *mpk6* mutant is a T-DNA insertion line (SALK_073907). Homozygous mutant plants were identified by PCR with *MPK3*-, *MPK6*-, or *MKK7*-specific primers (S4A–S4C Fig and S1 Table). In vivo kinase assays were unable to detect phosphorylated MPK3 and MPK6 in their respective homozygous double mutants (Fig 1B). In addition, the expression levels of *MKK7* in the double mutants of *mpk3bud1* and *mpk6bud1* were as high as those in *bud1* (S4D Fig). Therefore, these two homozygous double mutants were used for further studies.

**Fig 2:**
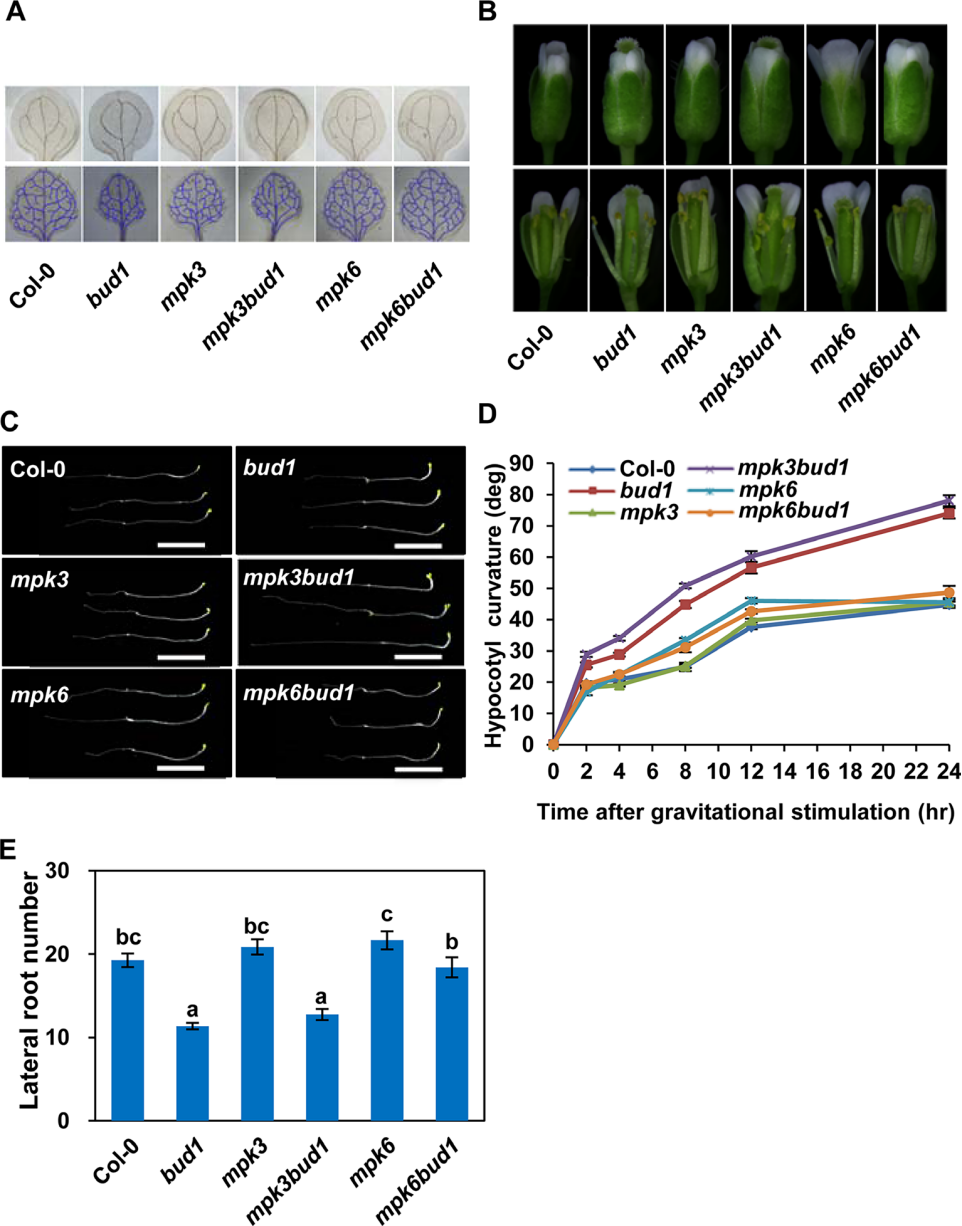
Genetic and morphological analysis of Col-0, *bud1*, *mpk3*, *mpk3bud1*, *mpk6*, and *mpk6bud1* plants. (A) Vascular systems of cleared specimens of Col-0, *bud1*, *mpk3*, *mpk3bud1*, *mpk6*, and *mpk6bud1* plants. The seedlings grown on MS plates for 12 d were taken with the same magnification. The upper panel refers to the vascular system of cotyledon and the lower to leaves. (B) The filament elongation of Col-0, *bud1*, *mpk3*, *mpk3bud1*, *mpk6*, and *mpk6bud1* flowers grown under long day conditions. (C) Gravitropic responses of dark-grown seedlings. Seedlings were grown in the dark for 4 d. The plates were reoriented by 90° and photographed after 18 h of gravistimulation. Bars, 0.5 cm. (D) Kinetic analysis of hypocotyl gravitropism. Seedlings were grown in the dark for 3 d on 0.5 × MS plates and reoriented by 90°. The gravitropic curvatures were measured at the time as indicated. Values are means ± SE (*n* = 20). (E) Statistical analysis of lateral root number. Lateral root numbers were counted at 12 d after germination. The values are means ± SE (*n* = 19). According to Turkey’s honest significant difference (HSD) test (*p* < 0.05), means of lateral root number do not differ if they are indicated with the same letter.

**Fig 3:**
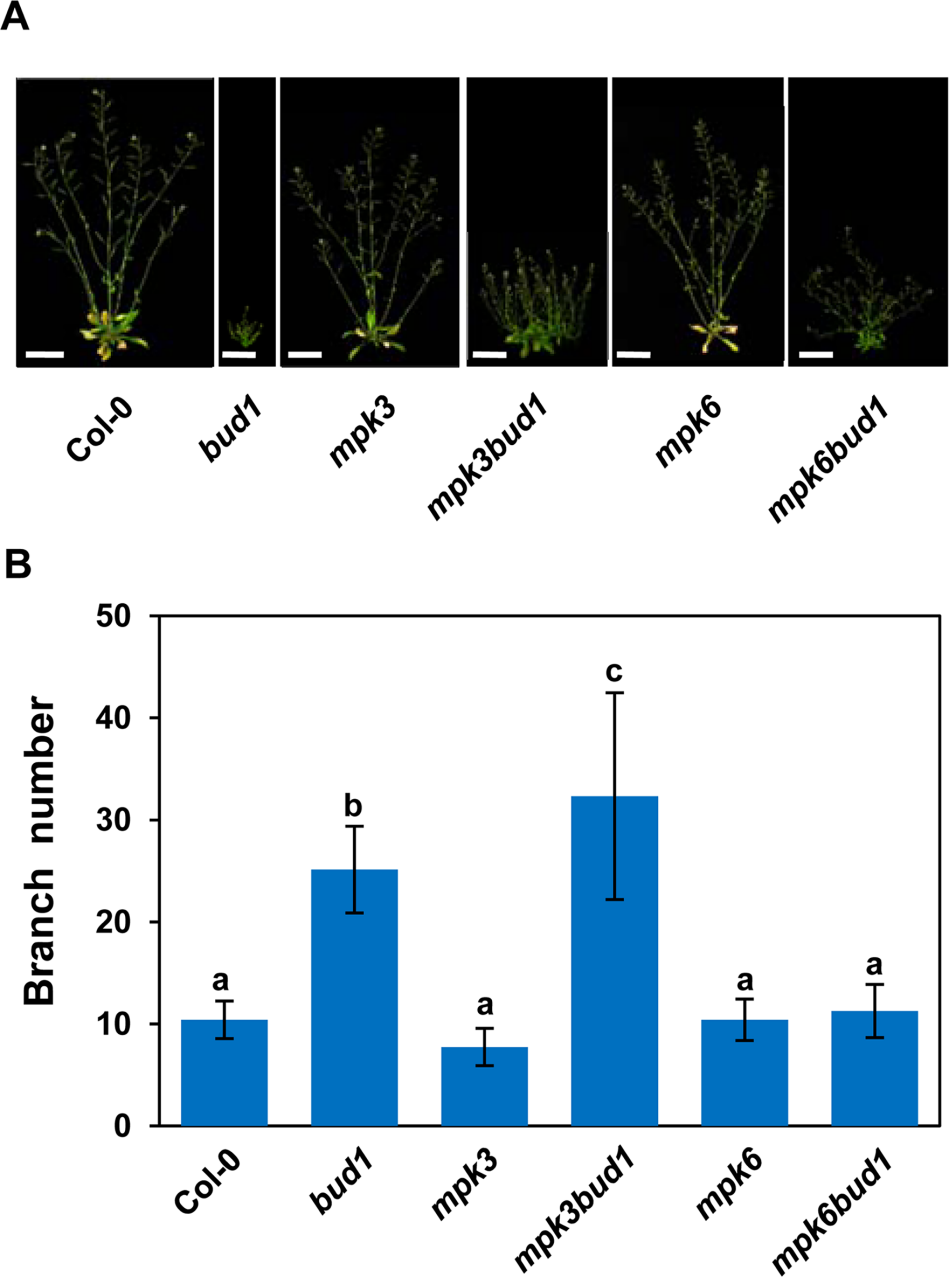
The MKK7-MPK6 cascade is involved in shoot branching. (A) Branching phenotypes of 50-d-old Col-0, *bud1*, *mpk3*, *mpk3bud1*, *mpk6*, and *mpk6bud1* plants grown under the long day condition. Bar, 5 cm. (B) The branch numbers of 50-d-old plant. Each value represents the mean ± SD (*n* = 15). According to Turkey’s HSD test (*p* < 0.05), means of branch number do not differ if they are indicated with the same letter.

To determine the contribution of MKK7-MPK3 and MKK7-MPK6 cascades for the multiple phenotypes in *bud1*, we first compared the phenotypes among the wild-type, *bud1*, *mpk3*, *mpk6*, *mpk3bud1*, and *mpk6bud1* plants. The results showed that the leaf venation pattern (Fig 2A), filament elongation (Fig 2B), gravitropism of dark-grown seedlings (Fig 2C and 2D), lateral root number (Fig 2E and S5 Fig), and branch number in the *mpk6bud1*double mutant were restored to the wild-type, whereas these phenotypes in the *mpk3bud1* double mutant were similar to those in the *bud1* mutant (Fig 3). On the other hand, the curled leaves in the *bud1* mutant were largely rescued in *mpk3bud1* plants and partially rescued in *mpk6bud1* plants (S6 Fig). Genetic analysis indicated that the MKK7-MPK6 cascade is specifically involved in multiple aspects of plant development including the regulation of leaf venation architecture, gravitropism, filament elongation, lateral root formation, and shoot branching, whereas the MKK7-MPK3 and MKK7-MPK6 cascades function redundantly in leaf morphology.

### The MKK7-MPK6 Cascade Regulates PAT in Shoots

**Fig 4:**
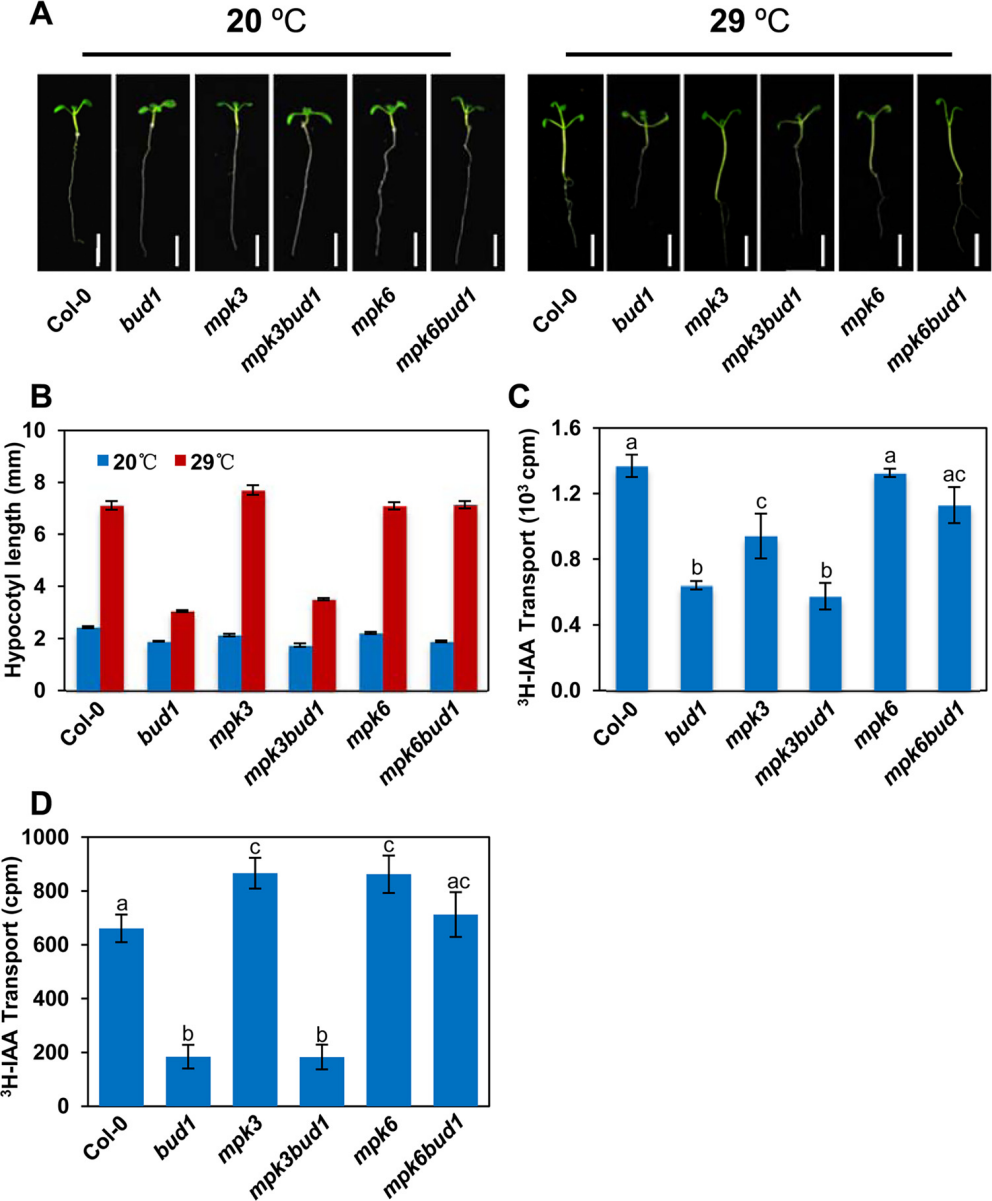
The MKK7-MPK6 cascade is involved in polar auxin transport. (A) Induction of hypocotyl elongation by high temperature. Wild-type and mutant seedlings were grown on 0.5 × MS solid media at 20°C and 29°C, respectively, and photographed at 9 d after germination. Bars, 5 mm. (B) Statistical analysis of high temperature-induced hypocotyl elongation. Values are means ± SE (*n* = 20). (C) Polar auxin transport assays of seedlings. Values are means ± SE of three independent assays. The difference significance was determined with Turkey’s HSD test (*p* < 0.05). (D) Polar auxin transport assays of inflorescence stems. Values are means ± SE of ten independent assays. The difference significance was determined with Turkey’s HSD test (*p* < 0.05).

The *bud1* mutant has been shown to exhibit the polar auxin transport (PAT) deficiency due to the overexpression of *MKK7* [34]. Because PAT is highly related to shoot development [41,42], we therefore speculated that the MKK7-MPK6 cascade may regulate shoot development through affecting PAT. To verify this speculation, we first examined the hypocotyl elongation of the wild-type, *bud1*, *mpk3*, *mpk6*, *mpk3bud1*, and *mpk6bud1* plants under a high temperature condition. Abnormal hypocotyl elongation upon a high temperature treatment indicates the deficiency in auxin-related pathways [43]. As previously reported, when grown in the light at high temperature (29°C), the wild-type seedlings exhibited dramatic hypocotyl elongation compared with the seedlings grown at 20°C, whereas the hypocotyl of *bud1* could not elongate under 29°C [34]. Our present study showed that this temperature-dependent growth response of the *mpk6bud1* hypocotyl was comparable to that of the wild-type, whereas *mpk3bud1* hypocotyl was still similar to that of the *bud1* mutant (Fig 4A and 4B), indicating that the PAT deficiency of *bud1* is due to the constitutive activation of MKK7-MPK6 cascade. We then measured the PAT in the hypocotyl segments of light-grown seedlings. To measure basipetal movement of ^3^H-indole-3-acetic acid (^3^H-IAA), a single microdroplet was applied to the apex of 4.5-d-old, light-grown seedlings. The auxin transport in the *bud1* hypocotyl segments was reduced to 46.7% of that of the wild-type, and *mpk3bud1* double mutants still showed the similar level as that in *bud1* mutants, although it was restored to 82.5% of that of the wild type in *mpk6bud1* double mutants (Fig 4C).

We extended our assay to measure the inflorescence stem basipetal PAT of wild-type, *bud1*, *mpk3*, *mpk3bud1*, *mpk6*, and *mpk6bud1* plants. As shown in Fig 4D, the auxin basipetal transport in *bud1* and *mpk3bud1* was significantly reduced to 28% of that of the wild type, whereas in the *mpk6bud1* inflorescence stem, auxin basipetal transport was restored to a comparable level to that of the wild type.

Taken together, these data demonstrated that it is the MKK7-MPK6 but not MKK7-MPK3 cascade that is responsible for PAT in shoots.

### Change of the PAT Correlated to Auxin Distribution

To test whether the change of the PAT is correlated to auxin distribution in the main stem, we visualized the auxin distribution by DR5-green fluorescent protein (DR5-GFP). Our results showed that, unlike in the wild-type plants, *DR5* activity can be barely detected in *bud1* inflorescence stems, whereas the *mpk6bud1* double mutant showed similar *DR5* activity to that in wild-type plants; however, significantly decreased *DR5* activity was observed in *mpk3bud1*, which was comparable to the *bud1* mutant (S7 Fig). These results demonstrate that the MKK7-MPK6 cascade is involved in PAT and has a direct impact on the auxin distribution in inflorescence stems.

### The MKK7-MPK6 Cascade Regulates the PIN1 Polar Localization

**Fig 5:**
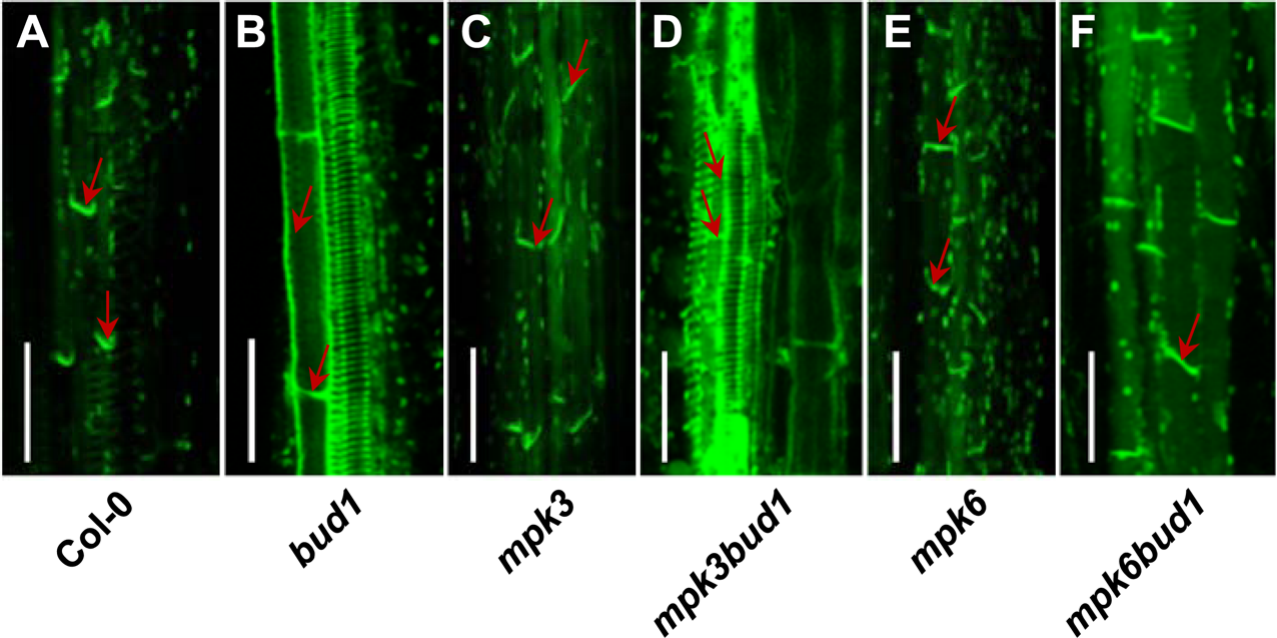
The MKK7-MPK6 cascade regulates PIN1 polar localization in shoot stem. Localization of PIN1-GFP in longitudinal hand sections of 35-d-old basal inflorescence stems of Col-0 (A), *bud1* (B), *mpk3* (C), *mpk3bud1* (D), *mpk6* (E), and *mpk6bud1* (F). Sections were mounted in water, and the GFP signal was examined under a confocal microscope at an excitation wavelength of 488 nm. Red arrows indicate PIN1-GFP localization. Bars, 50 μm.

The *Arabidopsis* PIN1 protein is basally localized in stem xylem parenchyma cells, where it is required for auxin transport [44]. To determine whether the MKK7-MPK6 cascade regulates PAT through PIN1, *PIN1pro*::*PIN1-GFP* plants were crossed into *bud1*, *mpk3*, *mpk3bud1*, *mpk6*, and *mpk6bud1* mutants, respectively. The inflorescence stems of 30-d-old homozygous plants were used for the analysis of PIN1-GFP localization. Unlike the wild-type, *mpk3*, and *mpk6* plants, in which the PIN1-GFP is basally localized in xylem parenchyma cells, the *bud1* and *mpk3bud1* mutants did not show typical basal localization of PIN1-GFP (Fig 5A–5E), whereas PIN1-GFP in the *mpk6bud1* double mutants exhibited the typical basal localization in xylem parenchyma cells (Fig 5F), demonstrating that the MKK7-MPK6 cascade is responsible for regulating the PIN1-GFP basal localization in the inflorescence stem.

### The MKK7-MPK6 Cascade Phosphorylates PIN1 Directly In Vitro

**Fig 6:**
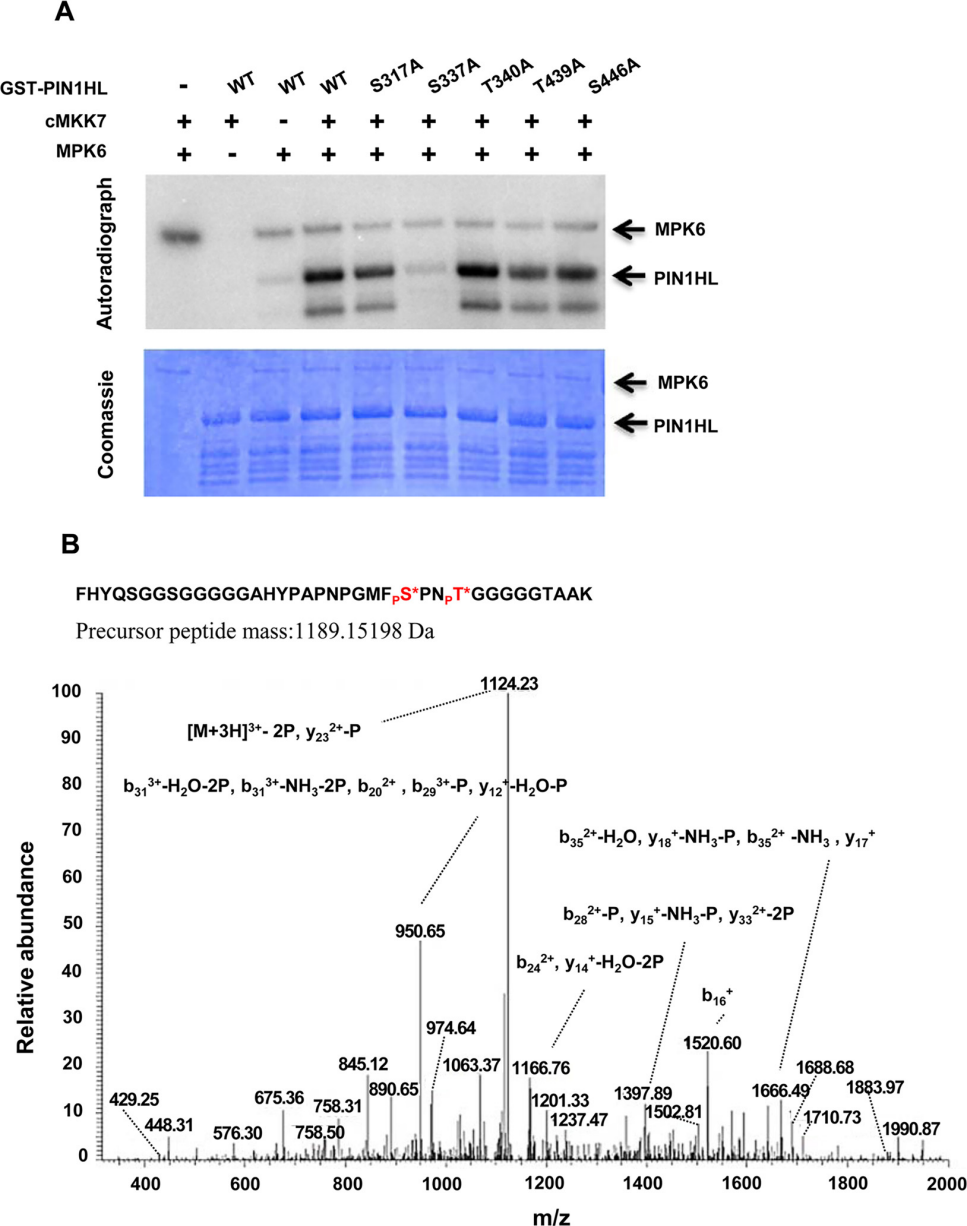
In vitro phosphorylation assay of PIN1HL by the MKK7-MPK6 cascade. (A) In vitro assay of phosphorylation by the MKK7-MPK6 cascade using wild-type GST-PIN1HL or site-mutated PIN1Hl, in which the indicated residues were replaced with Ala (A) residues, respectively. The positions of GST-PIN1HL are indicated in the autoradiograph (top panel) and the Coomassie-stained gel (bottom panel). The Coomassie blue-stained gel was used as a control for protein loading. (B) Spectra for representative identified phosphopeptides S337 and T340. Asterisk represents the phosphate moiety.

Evolutionary conserved phosphorylation sites within the central HL of PIN proteins were found to be essential for the apical and basal polar PIN localizations [32]. D6 PROTEIN KINASE (D6PK) and PID kinases belong to the *Arabidopsis* AGCVIII kinase family and regulate PIN1 localization through phosphorylating different sites [29,31,32,45]. Although several phosphorylation sites within the central HL of PIN proteins have been identified, only three phosphorylation sites (S231, S252, and S290) in the HL of PIN1 were verified to be the targets for PID and one phosphorylation site (S271) for D6PK phosphorylation [29,31,32,45], implying that protein kinases other than PID and D6PK may target PIN1 for phosphorylation. To verify whether the MKK7-MPK6 cascade may phosphorylate PIN1, we performed an in vitro protein kinase assay by incubating Glutathione S-transferase (GST)-tagged HL of PIN1 (PIN1HL), GST-tagged MPK6, and Histidine (HIS)-NusA-tagged cMKK7 in an in vitro phosphorylation reaction. As shown in Fig 6A, cMKK7-MPK6-dependent phosphorylation of GST-PIN1HL was detected. To further elucidate molecular mechanisms of PIN1 polar localization regulated by MKK7-MPK6 cascade phosphorylation, we identified the MKK7-MPK6 phosphorylation sites in the PIN1HL. First, we performed Liquid Chromatograph-Mass Spectrometer/Mass Spectrometer (LC-MS/MS) analysis to identify the phosphorylation sites. A total of 12 phosphorylation sites were detected in the PIN1HL (Fig 6B; S8 and S9 Figs), and five main phosphorylation sites (S317, S337, T340, T439, and S446) were selected for further analysis (Fig 6B and S8 Fig). These five sites showed more than 75% probabilities for the phosphorylation (S9 Fig) and are not conserved within the central HL of PIN proteins (S10 Fig). Next, we tested the effect of Ser/Thr-to-Ala substitution of these five sites (S317A, S337A, T340A, T439A, and S446A) on MKK7-MPK6 phosphorylation using GST-tagged PIN1HL. As shown in Fig 6A, a single S337A substitution led to a dramatic reduction of phosphorylation by MKK7-MPK6, whereas other single substitution could not alter the phosphorylation status of GST-tagged PIN1HL, indicating that S337 of PIN1HL is the dominant phosphorylation site by the MKK7-MPK6 cascade.

### Phosphorylation of PIN1 S337 Residue Directs Its Polar Localization and Determines Auxin-Regulated Shoot Branching

To investigate the biological significance of the S337 site of PIN1 *in planta*, various mutant constructs were generated from *35S*::*PIN1-GFP*, in which S337 in the encoded PIN1-GFP proteins was replaced by Ala (A), an nonphosphorylatable residue, or by Asp (D) to mimic phosphorylation. The resulting constructs *35S*::*PIN1*^*S337A*^*-GFP* and *35S*::*PIN1*^*S337D*^*-GFP* were respectively transformed into *Arabidopsis Columbia* (Col) wild-type plants. Western blot result showed that PIN1-GFP in *35S*::*PIN1*^*S337A*^*-GFP* and *35S*::*PIN1*^*S337D*^*-GFP* transgenic plants expressed as well as in *35S*::*PIN1*^*WT*^ transgenic plants (S11 Fig).

**Fig 7:**
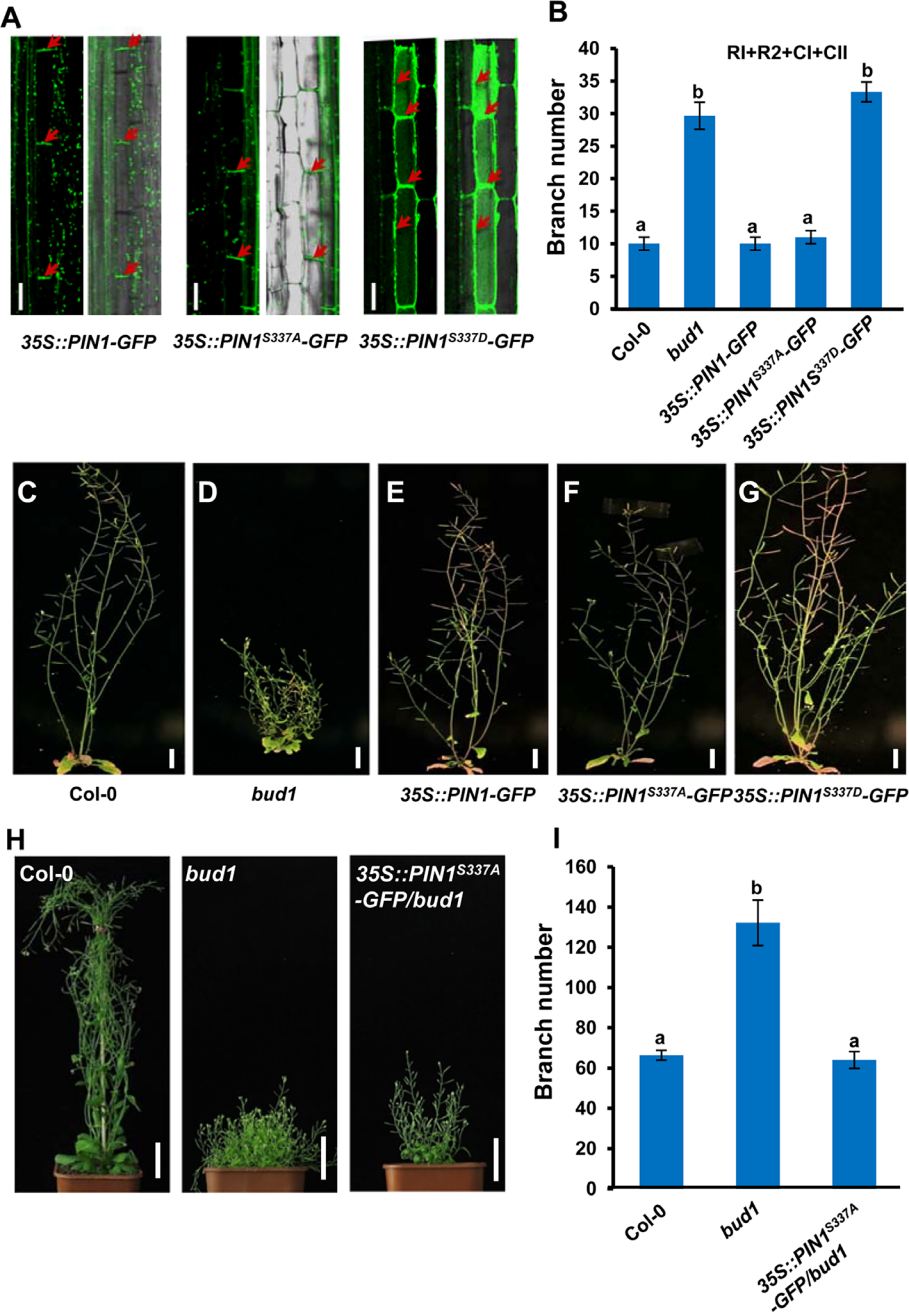
The S337 phosphorylation status is responsible for PIN1 polar localization and regulation of shoot branching. (A) PIN1-GFP localization in inflorescence stems of *35S*::*PIN1*^*WT*^*-GFP*, *35S*::*PIN1*^*S337A*^*-GFP*, and *35S*::*PIN1*^*S337D*^*-GFP* transgenic plants. Red arrows indicate PIN1-GFP localization. Bars, 50 μm. (B) Comparison of branch number among wild-type, *bud1*, *35S*::*PIN1*^*WT*^*-GFP*, *35S*::*PIN1*^*S337A*^*-GFP*, and *35S*::*PIN1*^*S337D*^*-GFP* transgenic plants. Primary rosette-leaf branch (RI), secondary rosette-leaf branch (RII), primary cauline-leaf branch (CI), and secondary cauline-leaf branch (CII) were counted at 60 d. Data are shown as mean ± SE (*n* ≥ 17). The difference significance was determined with Turkey’s HSD (*p* < 0.01). (C–G) Expression of phospho-mimicking *PIN1*^*S337D*^ confers branching phenotype (G), whereas the expression of nonphosphorylatable *PIN1*^*S337A*^ (F) or wild-type *PIN1* (E) results in normal phenotype. Pictures of representative transgenic lines, along with Col-0 and *bud1*, grown under long day conditions, were taken at 60 d. Bars, 5 cm. (H) Branching phenotypes of 50-d-old Col-0, *bud1*, and *35S*:*PIN1*^*S337A*^*/bud1* plants grown under the long day condition. Bar, 4 cm. (I) Branch number of 50-d-old plant. Data are shown as mean ± SE (*n* = 15). The difference significance was determined with Turkey’s HSD test (*p* < 0.01).

First, we examined whether the PIN1-GFP subcellular localization in *Arabidopsis* inflorescence stems is influenced by the phosphorylation status of S337. As shown in Fig 7A and S12A Fig, the subcellular localization of PIN1-GFP in *35S*::*PIN1*^*S337D*^*-GFP* transgenic plants failed to properly establish polarity in the xylem parenchyma cells, showing similar apolar localization of PIN1-GFP in *bud1* (Figs 5B and 7A and S12A Fig). By contrast, the polarity of PIN1-GFP in *35S*::*PIN1*^*S337A*^*-GFP* transgenic plants was not changed (Fig 7A), suggesting that S337 phosphorylation status is essential for PIN1 polarity in *Arabidopsis* inflorescence stem.

In addition, we performed a detailed analysis of PIN1-GFP localization in both roots and hypocotyls. As shown in S13 Fig, PIN1-GFP localization has no obvious difference in Col-0, *bud1*, *mpk3*, *mpk3bud1*, *mpk6*, and *mpk6bud1* roots. In hypocotyls, PIN1-GFP localizations in Col-0, *bud1*, *mpk3*, *mpk3bud1*, *mpk6*, and *mpk6bud1* are similar to those observed in inflorescence stems (S14A Fig). However, PIN1^S337D^-GFP exhibited typical basal localization similar to PIN1-GFP and PIN1^S337A^-GFP (S14B Fig). Consistent with this, the physiological analysis showed that the high temperature-dependent growth response in *35S*::*PIN1*^*S337D*^ hypocotyls was comparable to those in *35S*::*PIN1*^*WT*^ and *35S*::*PIN1*^*S337A*^ hypocotyls (S14C and S14D Fig). These results demonstrated that PIN1 phosphorylation by MKK7-MPK6 cascade is likely organ-specific.

To verify whether the altered PIN1 localization mediated by phosphorylation of S337 affects the shoot branching, we compared the branching phenotypes of the transgenic plants. The results showed that the branch number of *35S*::*PIN1-GFP* and *35S*::*PIN1*^*S337A*^*-GFP* transgenic plants were comparable to those of the wild type (Fig 7B–7F). However, the *35S*::*PIN1*^*S337D*^*-GFP* transgenic plant displayed more branches than the wild type (Fig 7G and S12B–S12F Fig), demonstrating that constitutive phosphorylation of the PIN1 S337 may contribute significantly to the branching phenotype of the *bud1* mutant.

To further understand the role of PIN1 phosphorylation on S337 by the MKK7-MPK6 cascade, we transformed the phospho-deficient version of PIN1 (*PIN1*^*S337A*^) in the *bud1* background. The homozygous T_3_ line in which *MKK7* expression level was as high as that in the *bud1* mutant was identified for further analysis (S15 Fig). The result showed that overexpression of *PIN1*^*S337A*^ in the *bud1* mutant could rescue the branching phenotype of *bud1* (Fig 7H and 7I), suggesting that the regulation of PIN1 polar localization through phosphorylation of S337 by the MKK7-MPK6 cascade is specific to the regulation of shoot branching.

**Fig 8:**
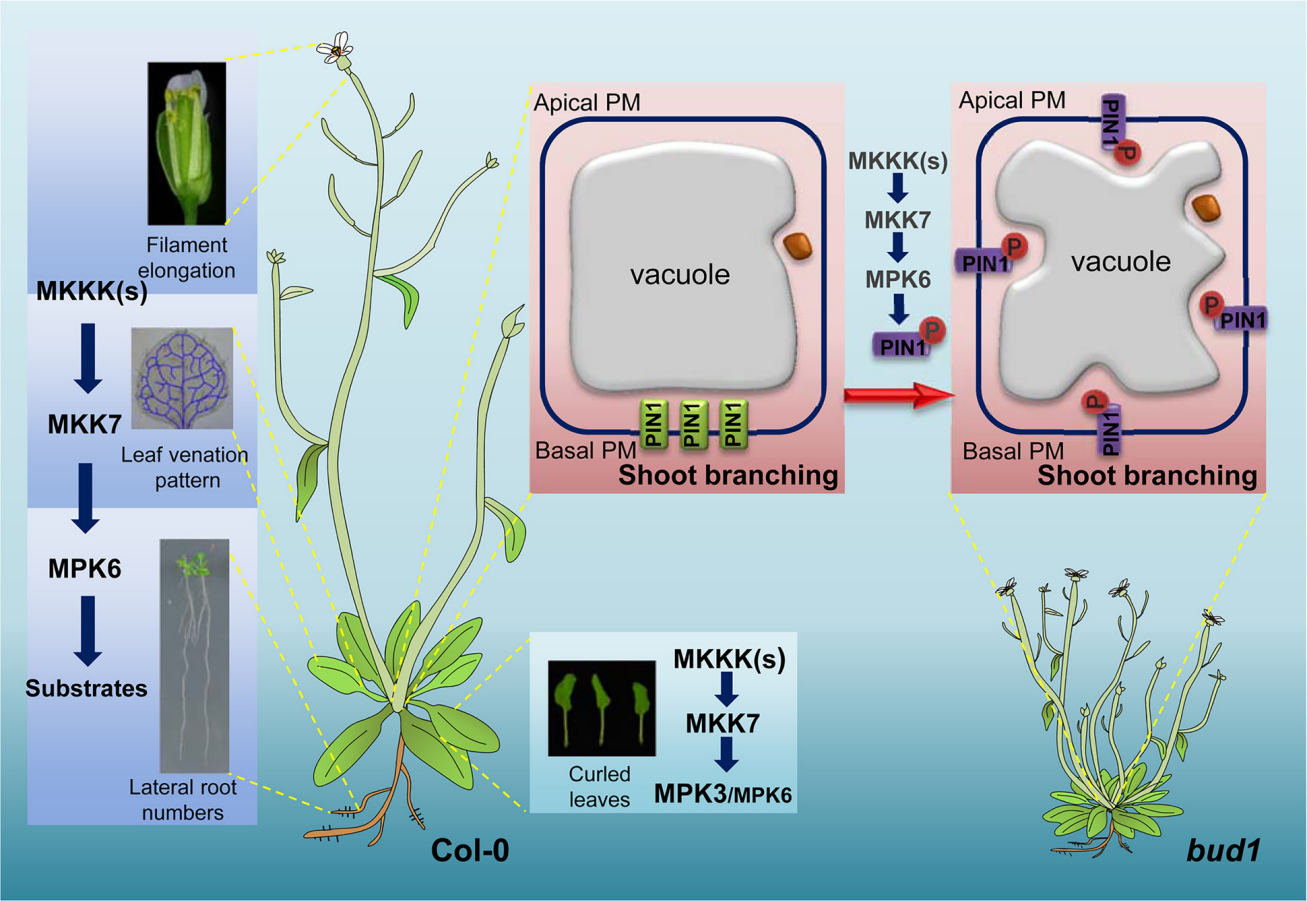
A proposed working model of the MKK7-MPK6/3 cascade involved in plant development in *Arabidopsis*. The MKK7-MPK6 cascade plays predominant roles in diverse developmental processes including leaf venation architecture, filament elongation, lateral root formation, and shoot branching. MKK7-MPK3 and MKK7-MPK6 cascades function redundantly in leaf morphology; the MKK7-MPK6 signaling pathway regulates PAT through phosphorylating PIN1. In the wild type, PIN1 basal localization is controlled by reversible phosphorylation of S337 site by the MKK7-MPK6 cascade. In the *bud1* plants, constitutively activated MKK7-MPK6 signaling leads to sustained phosphorylation of the PIN1 S337 site, which leads to PIN1 apolar localization and results in branching phenotype.

Taken together, our results demonstrated that MPK6 and MPK3 make different contributions downstream of MKK7. The MKK7-MPK6 cascade plays predominant roles in diverse developmental processes, including leaf venation architecture, gravitropism, filament elongation, lateral root formation, and shoot branching, whereas the MKK7-MPK3 cascade mainly regulates leaf morphology in a coordinative manner with the MKK7-MPK6 cascade (Fig 8). Furthermore, we propose a new mechanism that the MKK7-MPK6 signaling pathway regulates PAT through phosphorylating PIN1 to determine shoot branching (Fig 8). In the wild type, PIN1 basal localization is controlled by reversible phosphorylation of the S337 site by the MKK7-MPK6 cascade, which in turn determines PIN1 polarity and auxin flow. In the *bud1* plant, constitutively activated MKK7-MPK6 signaling leads to sustained phosphorylation of the PIN1 S337 site, which disturbs the PIN1 polarity and auxin gradients and results in branching phenotype.

## Discussion

The MAPK signaling pathway participates in many fundamental and important biological processes in all eukaryotes [46]. One of the most intriguing and puzzling questions in the MAPK signaling pathway is the maintenance of the signaling specificity in the MAPK cascades. In this study, we demonstrated that the MKK7-MPK6 cascade specifically regulates plant development. In particular, the MKK7-MPK6 cascade directly targets the PIN1 protein for phosphorylation at the S337 site to regulate shoot branching in *Arabidopsis*. Our results not only specify the functions of the MKK7-MPK6 cascade but also reveal a novel mechanism for PIN1 phosphorylation in shoot branching regulation.

### MKK7 Activates Two Downstream MAPKs to Perform Distinct Biological Functions

Previously, we identified a semidominant *Arabidopsis bud1* mutant, which results from the increased expression of *MKK7*, and demonstrated that MKK7 affects plant architecture by negatively controlling PAT, and the kinase activity of MKK7 is essential for its biological functions [34]. In addition, the *bud1* mutant also showed elevated levels of salicylic acid (SA), constitutive *pathogenesis-related* (*PR*) gene expression, and enhanced resistance to both *Pseudomonas syringae pv*. *maculicola* (*Psm*) *ES4326* and *Hyaloperonospora parasitica Noco2*, indicating that MKK7 positively regulates plant basal and systemic acquired resistance [47]. These studies shown above suggested that MKK7 may regulate plant development and resistance through activating different downstream MPKs.

In this study, we found that MKK7 can phosphorylate MPK3 and MPK6 in vivo, and the MKK7-MPK6 and MKK7-MPK3 cascades perform distinct functions *in planta*. The MKK7-MPK6 module mainly contributes to plant growth and development, which includes shoot branching (Fig 3), venation pattern of both cotyledon and true leaves (Fig 2A), filament elongation (Fig 2B), hypocotyl gravitropism (Fig 2C and 2D), and lateral root development (Fig 2E and S5 Fig). However, the MKK7-MPK3 module contributes little to plant development. Given that MKK7 is also involved in plant basal and systemic acquired resistance, we suggest that the MKK7-MPK3 may contribute mainly to defense response.

### Regulation of Auxin-Mediated Shoot Branching by MKK7-MPK6-Dependent Phosphorylation of PIN1

PAT is important for the establishment of auxin gradients, which are essential for plant polar growth and morphological patterning [48,49]. Although different PIN proteins contributing to intercellular and intracellular auxin transports have been reported previously, the roles of PINs in PAT-mediated shoot branching remain unclear. Previous studies revealed that PIN1 phosphorylation serves a key role in regulating PAT and auxin-related plant development [50,51]. For example, PID and PP2A antagonistically regulate PIN1 phosphorylation and mediate PIN1 apical-basal polar targeting in roots and shoots apex in *Arabidopsis* [31]. Further study showed that S231, S252, and S290 of PIN1 were the PID-dependent phosphorylation sites, and phospho-mimicking substitutions at these three sites induce apical localization of PIN1 [29]. PID belongs to a large family of AGCVIII kinases in *Arabidopsis* [52,53]. D6 PROTEIN KINASES (D6PK), another subfamily of AGCVIII kinases, mainly targets S271 instead of S231, S252, and S290 of PIN1 to control its activation and polar distribution [45]. It is suggested that S271 is a novel PIN1 protein phosphosite with a role in promoting auxin efflux [45]. In addition, PIN1 phosphorylation at S337 and T340 mediates its polarity and auxin distribution as well [32]. Recently, a study showed that a peptidyl-prolyl *cis/trans* isomerase Pin1At effect on PIN1 subcellular localization is mediated by PIN1 phosphorylation at S337/T340 [54]. However, both of these two sites are not directly phosphorylated by AGCVIII kinases in vitro, indicating that S337 and T340 phosphorylation sites could be a target for other potential kinases [32]. In this study, we demonstrated that the MKK7-MPK6 cascade could phosphorylate PIN1 at S337 (Fig 6), which affects PIN1 polar localization in xylem parenchyma cells (Fig 7A and S12A Fig). Overexpression of phospho-mimicking *PIN1*^*S337D*^ resulted in more branching phenotype (Fig7G and S12B–S12F Fig). However, the other phenotypes, such as plant height, filament elongation, hypocotyl gravitropism, and lateral root development, have not emerged in overexpression of phospho-mimicking *PIN1*^*S337D*^ transgenic plants. In addition, overexpression of *PIN1*^*S337A*^ in *bud1* mutant resulted in significantly reduced branch number; however, the other phenotypes of *bud1* have not been rescued (Fig 7H and 7I). Based on these results, we proposed that the regulation of PIN1 polar localization through reversible phosphorylation of S337 residue of PIN1 by the MKK7-MPK6 cascade is an essential mechanism that might be specific to shoot branching regulation.

Moreover, the previous report showed that MPK6 localized to the cytosol, nucleus, and the plasma membrane [55]. We further examined the subcellular localization of MKK7-GFP and MPK6-GFP fusion proteins in tobacco epidermal cells and verified that neither MKK7-GFP nor MPK6-GFP showed polar localization (S16 Fig). Considering that PIN1 S337 within HL has a presumably cytoplasmic orientation, we speculated that phosphorylation of PIN1 S337 by the MKK7-MPK6 cascade most likely occurs in the cytosol. However, much more work needs to be done to elucidate where MPK6 regulates the PIN1 in the cell.

### MAPKs and PIN Proteins: New Partners in PAT-Regulated Plant Development

Multiple phosphorylation sites in PIN proteins, of which some are targets of ACGVIII kinases, mediate PIN polarity [32]. Therefore, identification of the upstream components in the phosphorylation cascade is a big challenge [32]. Among all PIN1 phosphorylation sites, S337 and T340 are in the MFSPNTG sequence. As MAPKs preferably phosphorylate Ser or Thr residues followed by a Pro [56], the speculation that S337 might be a target of MAPKs was proposed nearly ten years ago [57]. However, until now, there has been no direct evidence to support this hypothesis. Our results provided a solid genetic and cytological evidence to reveal an important role of the MKK7-MPK6 cascade-dependent PIN1 phosphorylation at S337 in controlling shoot branching. Here, we identified another new upstream component of PIN1 phosphorylation in addition to the well-studied ACGVIII kinases, establishing a novel relationship between a specific MAPK pathway and PIN1 polarity and its function in regulating shoot branching.

Based on these studies, PIN proteins can be phosphorylated by at least three different protein kinases: D6PKs, PID, and MAPKs. PID-dependent PIN1 phosphorylation functions in early plant development stages, such as embryo development and organogenesis [29,31], while our result showed that PIN1 plays an important role downstream of the MKK7-MPK6 cascade in regulating PAT-mediated shoot branching. Moreover, it has been shown that D6PKs and PID have different functions in the control of PIN3-mediated phototropic bending [58], suggesting that these three different kinases regulate PIN-mediated auxin transport at the temporal and spatial level, which implies that spatial and temporal regulation of PIN polarity might be partly attributed to the specific phosphorylation sites of ACGVIII kinases and MAPKs.

Another possibility that MAPKs or ACGVIII kinases maintain spatio-temporal feature is to phosphorylate specific PIN(s) in a given biological pathway. In our study, the MKK7-MPK6 cascade mainly regulates PIN1-mediated auxin transport in shoot. However, *bud1* has other phenotypic defects such as plant height, the hypocotyl gravitropism, filament elongation, and lateral root development (Fig 2). This prompts us to speculate that these phenotypes of *bud1* are the consequence of defective auxin transport activity caused by other PINs, such as PIN2, PIN3, PIN4, or PIN7. It was reported that phosphorylation status of PIN3 plays a decisive role in root gravitropism [59]. Our alignment analysis also showed that S337 of PIN1 is conserved in PIN3, which is corresponding to S317 site of PIN3 (S10 Fig), implying that the MKK7-MPK6 cascade might regulate hypocotyl gravitropism by phosphorylating the S317 site of PIN3. However, five main predicted phosphorylation sites (S317, S337, T340, T439, and S446) are not conserved within the central HL of PIN proteins (S10 Fig). Thus, finding the major phosphorylation site by MAPK on other PINs will help to further establish the relationship between a specific MAPK pathway and PIN polarity during plant development.

## Materials and Methods

### Plant Materials

*Arabidopsis thaliana* plants were grown on the mixture of vermiculite and soil (2:1) saturated with 0.3 × B5 medium under long day condition (80–120 μE m^-2^ s^-1^) at 22°C. For plants grown in Petri dishes, seeds were surface sterilized with 70% (v/v) ethanol for 3 min and 12% (v/v) commercial bleach solution for 15 min, rinsed 5 times with sterile water, and suspended in 0.2% agar. The sterilized seeds were plated on 0.5 × Murashige & Skoog (MS) medium containing 0.8% agar and pre-incubated at 4°C in the dark for 3 d before being cultured under the conditions as indicated. For temperature treatment, plants were germinated and grown on 0.5 × MS medium in versatile environmental test chambers (Sanyo) under continuous illumination at 20 and 29°C, respectively.

### The Generation of the *mpk3bud1* and *mpk6bud1* Double Mutants

The *mpk3* mutant results from a deletion of 6.3 kb fragment by fast neutron mutagenesis [40], and the *mpk6* mutant (SALK_073907) is a T-DNA insertion mutant obtained from ABRC. The double mutants were generated from the cross of homozygous *bud1* with *mpk3* and *mpk6*, respectively, and identified by PCR-based method (S4A–S4C Fig) [34]. All the genotyping primers can be found in S1 Table.

### Identification of the *MKK7* Knockout Mutant

The *mkk7* mutant (CS110477) obtained from ABRC results from transposon insertion. Homozygous mutant plants were identified by PCR with *MKK7*-specific primers (S2A Fig). RT-PCR data showed that *mkk7* did not produce detectable *MKK7* RNA (S2A Fig). The primers for genotyping were listed in S1 Table.

### Polar Auxin Transport Assay

Auxin transport from the shoot apex into roots in seedlings was conducted using intact light-grown seedlings as described previously [34] with the following modifications: seedlings used in this assay were grown on 0.6% phytagel (SIGMA, P-8169) plates containing 0.25 × MS (pH 5.8) and 0.5% sucrose. Seedlings were grown 4.5 d after germination. Before assay, 10 seedlings were transferred to vertically discontinuous filter paper strips saturated in one-quarter MS medium and allowed to equilibrate for at least 2 h. Auxin solution used to measure transport was made up in 0.25% agarose containing 25 mM MES (2-[N-Morpholino] ethanesulfonic acid), pH 5.2. A 0.2 μl microdroplet containing 500 nM unlabeled IAA or 500 nM ^3^H-IAA (specific activity 26 Ci/mmol) was placed on the shoot apical tip of seedlings using a 0.5 μl glass syringe. Seedlings were incubated in the dark for 6 h. After incubation, the hypocotyls and cotyledons were removed. A 2 mm section of filter paper, upon which the S-R TZ was centered, was harvested along with the 2 mm segment of tissue containing the S-R TZ. The samples were allowed to immerge in 2 ml of universal scintillation fluid for at least 18 h before being counted in a liquid scintillation counter.

Auxin transport in inflorescence stems was detected using 5-wk-old plants as described [60]. Stem segments, 2.5 cm in length cut under the first silique, were placed in 0.5 ml microcentrifuge tubes in inverted orientation and submerged in 20 μl of radioactive IAA solution (100 nM ^3^H-IAA in 0.05% MES, pH 5.5) at 22°C in darkness for 18 h. The base of the inflorescence submerged was used to measure background IAA movements. After incubation, a 5 mm section was excised from the nonsubmerged end of the segment and was transferred into 1 ml scintillation fluid. The samples were counted in a liquid scintillation counter after 18 h.

### Expression and Purification of Recombinant Proteins

The ORFs of MPKs were cloned into pET-28a vector (Novagen), and cMKK7 was cloned into pET-44a vector (Novagen) for recombinant protein expression. Fifteen MPKs were successfully expressed, and the other five MPKs (MPK11, 13, 15, 16, 18) failed to be expressed. The primers used for construction of recombinant proteins are listed in S2 Table. PIN1HL was cloned into the pET-60-DEST vector (Merck), and Quickchange XL site-directed mutagenesis kit (Stratagene) was employed to generate mutant constructs. The primers used for mutated PIN1HL are listed in S3 Table. Recombinant proteins were expressed and purified according to the manufacturer’s protocols, and the protein concentration was determined by Bio-Rad protein assay reagent. The recombinant proteins were confirmed by western blot using anti-GST and anti-HIS antibodies.

### In Vitro Kinase Assay

The in vitro kinase assay was carried out as previously reported with minor modifications [61]. The recombinant MPKs (2 μg) and cMKK7 (0.5 μg) were incubated in 20 μl kinase reaction buffer (50 mM Tris-HCl pH 7.5, 10 mM MgCl_2_, 10 mM NaCl, 0.1 mM ATP, and 1 mM DTT) at room temperature for 30 min. Then MBP (2 μg) and 3 μCi [γ-^32^P] ATP (3000 Ci/mmol, GE Health) were added for another 30 min incubation at room temperature. The phosphorylated MBP and MPKs were visualized by Typhoon Trio after being separated by 15% (w/v) SDS-PAGE.

### In Vitro Phosphorylation Assay of PIN1HL

The in vitro phosphorylation assay was conducted as previously described [61]. Recombinant GST-tagged MPK6 was firstly activated by recombinant cMKK7 in the kinase reaction buffer at the presence of 50 μM ATP at 22°C for 30 min. Then, activated MPK6 (20:1 substrate enzyme ratio) was incubated with GST-PIN1HL or GST-PIN1HL with substitution of various phosphorylation sites in the kinase reaction buffer with 25 μM ATP and 3 μCi [γ-^32^P] ATP (3,000 Ci/mmol) at 22°C. The reactions were stopped by adding SDS loading buffer after 60 min. Phosphorylated PIN1HL was visualized by autoradiography after being resolved in a 10% (w/v) SDS-PAGE gel.

### Microscopic Observation of PIN1-GFP

*PIN1pro*::*PIN1-GFP* plants were crossed into *bud1*, *mpk3*, *mpk3bud1*, *mpk6*, and *mpk6bud1* mutants, respectively, and homozygous lines were used for further analysis. Longitudinal hand sections were made from basal internodes of inflorescence stems (approximately 1 cm above the rosette) of 30-d-old plants. Sections were mounted in water, and GFP fluorescence was immediately observed on an OLYMPUS FV 1000 confocal laser scanning microscope. For each genotype, at least 20 samples were examined.

Agarose sectioning was employed to visualize PIN1-GFP in *35S*::*PIN1-GFP* transgenic lines. Briefly, inflorescence stems, approximately 1 cm above the rosette, were embedded in agarose (7% low melting agarose). After cooling in 4°C for 15 min, the segments coated by agarose were sliced with the Leica VT1200S in longitudinal direction at the thickness of 110 μm. The following microscopic observation procedure is the same as *PIN1pro*::*PIN1-GFP* transgentic plants.

### Microscopic Observation of DR5-GFP

*DR5-GFP* plants were crossed into *bud1*, *mpk3*, *mpk3bud1*, *mpk6*, and *mpk6bud1* mutants, respectively, and homozygous lines were used for further analysis. Transverse cross-sections of basal internodes of inflorescence stems were employed. Material preparation and GFP fluorescence detection were as described above.

### Transient Expression in *Nicotiana benthamiana*

Agrobacterium tumefaciens strain *EHA10*5 harboring binary vectors in 5 ml of Luria-Bertani (LB) medium with appropriate antibiotics was cultured overnight.

Bacteria solution was prepared and infiltrated into the 4-wk-old tobacco leaves as described [16]. Two to three d after the infiltration, the leaf disks were excised and mounted onto slides for confocal imaging. GFP fluorescence detection were as described above.

### Phosphopeptide Identification

The reaction mixture was lyophilized and reconstituted with 8 M urea in 25 mM NH_4_HCO_3_ (pH 7.4). The proteins were then digested with trypsin as previously described with slight modifications [62]. Briefly, the proteins were reduced with 10 mM DTT at 37°C for 4 h and alkylated with 25 mM iodoacetamide at room temperature for 1 h in the dark; in-solution trypsin digestion was performed at 37°C for 18 h using a trypsin:substrate ratio 1:50. The phosphopeptides were enriched by immobilized metal affinity chromatography (IMAC) using a well-established protocol [63] and then analyzed by LC-MS/MS using LTQ-Orbitrap elite mass spectrometer with enabled multistage activation. Phosphopeptides were identified by searching the International Protein Index database (IPI, *Arabidopsis*, version 3.85) using the software Proteome Discoverer (version 1.3). The phosphorylation probability was analyzed according to method as described in previous report [64].

### Transgenic Constructs

*PIN1-GFP* was amplified from *PIN1pro*::*PIN1-GFP* marker line [50] genomic DNA and then was cloned into the pB2GW7,0 vector (Ordered from the Department of Plant Systems Biology at Ghent University, Belgium). PIN1-GFP S337A and S337D substitutions were made as PIN1HL site mutations described above on entry vector. The *35S*::*MPK6-GFP* and *35S*::*MKK7-GFP* constructs were made by inserting *MPK6-GFP* and *MPK7-GFP* between the BamHI and EcoRI sites of binary vector PBI121, respectively. All the primers used for above cloning are shown in S4 Table.

### Real-Time PCR

Total RNA were isolated from aerial parts of 3-wk-old plants using a TRIzol kit (Invitrogen) according to the user manual. Real-time PCR was performed as described [65] and using primers listed in S5 Table.

## Supporting Information

S1 Data List of all individual quantitative data values that underlie the data summarized in the corresponding figure panel. (XLSX)

S1 Fig Screening for candidate substrates of MKK7 in vitro. In vitro kinase assays for screening candidate substrates of MKK7 by constitutively activated MKK7 (cMKK7). The cMKK7 was incubated with various MPKs in the kinase reaction buffer, respectively. Aliquots of the samples were separated by SDS-PAGE and subjected to autoradiography.
(TIF)

S2 Fig Identification and characterization of the *mkk7* mutant. (A) Identification of the *mkk7* mutant. Upper panel shows the diagram of *MKK7* and the transposon insertion site. Lower panel showing PCR and RT–PCR analysis confirmed the absence of *MKK7* in homozygous *mkk7*. (B) Branching phenotypes of 40-d-old Col-0, *bud1*, and *mkk7* plants grown under the long day condition. Total branches were counted at 40 d. Data are shown as mean ± SE (*n* = 15). The difference significance was determined with Turkey’s HSD test (*p* < 0.01). (C) Plant height of 30-d-old Col-0, *bud1*, and *mkk7* plants grown under the long day condition. Data are shown as mean ± SE (n = 16). The difference significance was determined with Turkey’s HSD test (*p* < 0.05). (D) Root architecture of Col-0, *bud1*, and *mkk7* seedlings. Plants were grown vertically on 0.5 × MS containing 1% sucrose and 0.6% phytagel plates and photographed at 11 d after germination (left panel). Bars, 1 cm. Statistical analysis of lateral root number (right panel). Data are shown as mean ± SE (*n* = 38). The difference significance was determined with Turkey’s HSD test (*p* < 0.01). (E) Polar auxin transport assays of inflorescence stems. Values are means ± SE of six independent assays. The difference significance was determined with Turkey’s HSD test (*p* < 0.01). (F) Induction of hypocotyl elongation by high temperature. Wild-type and mutant seedlings were grown on 0.5 × MS solid media at 20°C and 29°C, respectively, and photographed at 9 d after germination. Bars, 10 mm. (G) Statistical analysis of high temperature-induced hypocotyl elongation. Values are means ± SE (*n* ≥ 22). ** differences for the mutants compared with wild-type are highly significant (*p* < 0.01).
(TIF)

S3 Fig Alignment of MPK3 and MPK6. Analysis was performed by ClustalW2 online and edited by BioEdit software.
(TIF)

S4 Fig Molecular characterization of *mpk3bud1* and *mpk6bud1* homozygous lines. (A) Diagram showing binding sites of *bud1*genotyping primer. (B) Genotyping of the *mpk3bud1* double mutant. (C) Genotyping of the *mpk6bud1* double mutant. (D) Northern blot analysis showing the expression levels of *MKK7*, *MPK3*, and *MPK6* in Col-0, *BUD1/bud1*, *bud1*, *mpk3*, *mpk3bud1*, *mpk6*, and *mpk6bud1* plants.
(TIF)

S5 Fig Root architecture of Col-0, *bud1*, *mpk3*, *mpk3bud1*, *mpk6*, and *mpk6bud1* seedlings. Plants were grown vertically on 0.5 × MS containing 1% sucrose and 0.6% phytagel plates and photographed at 12 d after germination. Bars, 1 cm.
(TIF)

S6 Fig Leaf morphologies of Col-0, *BUD1/bud1*, *bud1*, *mpk3*, *mpk3bud1*, *mpk6*, and *mpk6bud1* plants. Bar, 2 cm.
(TIF)

S7 Fig The MKK7-MPK6 cascade may be involved in auxin distribution. DR5-GFP activity in basal stem segments of Col-0, *bud1*, *mpk3*, *mpk3bud1*, *mpk6*, and *mpk6bud1*. Bars, 100 μm.
(TIF)

S8 Fig Spectra for representative identified phosphopeptides of PIN1HL. (A) Spectra for representative identified phosphopeptide S317. Asterisk represents the phosphate moiety. (B) Spectra for representative identified phosphopeptide T439 and S446. Asterisk represents the phosphate moiety.
(TIF)

S9 Fig Prediction of the phosphorylation sites of PINIHL. The phosphopeptides were analyzed using LTQ-Orbitrap elite with enabled multistage activation, and the phosphosites were assigned by the software protein discovery. The amino acid sequence of PINIHL mapped with the identified phosphosites and the confidence of each phosphosite (probability) was shown, respectively, with the indicated color.
(TIF)

S10 Fig Alignment of the amino acid sequences of the HL of five *Arabidopsis* PIN proteins. The main phosphorylation sites of PIN1HL are marked by bright green. Residues that are conserved in all five PINHLs are indicated with dark gray.
(TIF)

S11 Fig Protein levels of PIN1-GFP in transgenic seedlings of *35S*::*PIN1*^*WT*^*-GFP*, *35S*::*PIN1*^*S337A*^*-GFP*, and *35S*::*PIN1*^*S337D*^*-GFP*. Total proteins were isolated from the same lines mentioned in Fig 7, and the PIN1-GFP protein levels were detected by immunoblotting with an anti-GFP monoclonal antibody. Rubisco staining was included for assessing equal protein loading.
(TIF)

S12 Fig The S337 phosphorylation-regulated PIN1 localization and shoot branching. (A) PIN1-GFP localization in inflorescence stems of *35S*::*PIN1*^*WT*^ and three independent 35S::*PIN1*^*S337D*^*-GFP* transgenic lines. Red arrows indicate PIN1-GFP localization. Bars, 50 μm. (B) Comparison of branch number in wild-type plants and three independent *35S*::*PIN1*^*S337D*^*-GFP* lines. Primary rosette-leaf branch (RI), secondary rosette-leaf branch (RII), primary cauline-leaf branch (CI), and secondary cauline-leaf branch (CII) were counted at 60 d. Data are shown as mean ± SE (*n* ≥ 17). The difference significance was determined with Turkey’s HSD test (*p* < 0.05). (C–F) Three transgenic lines harboring the phospho-mimicking *PIN1*^*S337D*^ display branching phenotype. Bars, 5cm.
(TIF)

S13 Fig PIN1-GFP polar localization in roots of Col-0, *bud1*, *mpk3*, *mpk3bud1*, *mpk6*, and *mpk6bud1*. Localization of PIN1-GFP in longitudinal sections of 7-d-old roots of Col-0, *bud1*, *mpk3*, *mpk3bud1*, *mpk6*, and *mpk6bud1*. Bars, 50 μm.
(TIF)

S14 Fig PIN1-GFP localization in hypocotyls. (A) Localization of PIN1-GFP in 7-d-old hypocotyls of Col-0, *bud1*, *mpk3*, *mpk3bud1*, *mpk6*, and *mpk6bud1*. Red arrows indicate PIN1-GFP localization. Bars, 50 μm. (B) PIN1-GFP localization in 7-d-old hypocotyls of *35S*::*PIN1*^*WT*^*-GFP*, *35S*::*PIN1*^*S337A*^*-GFP*, and *35S*::*PIN1*^*S337D*^*-GFP* transgenic plants. Red arrows indicate PIN1-GFP localization. Bars, 50 μm. (C) Induction of hypocotyl elongation by high temperature. Wild-type and mutant seedlings were grown on 0.5 × MS solid media at 20°C and 29°C, respectively, and photographed at 9 d after germination. Bars, 10 mm. (D) Statistical analysis of high temperature-induced hypocotyl elongation. Values are means ± SE (*n* ≥ 15).
(TIF)

S15 Fig qRT-PCR analysis of *MKK7* expression. Relative expression of *MKK7* in Col-0, *bud1*, and *35S*::*PIN1-GFP*^*S337A*^*/bud1* plants. *Actin2* and *Ubiquitin5* were used as an internal control and gene expression was normalized to the wild-type expression level. Data represent the average of three independent experiments ± SE.
(TIF)

S16 Fig Subcellular localizations of MKK7-GFP and MPK6-GFP proteins. Representative confocal images of MPK6-GFP and MKK7-GFP localizations in tobacco epidermal cells. Bars, 50 μm.
(TIF)

S1 Table Primers used for genotyping homozygous mutants. (DOC)

S2 Table Primers used for construction of recombinant proteins. (DOC)

S3 Table Primers used for construction of PIN1 site-directed mutagenesis. (DOC)

S4 Table Primers used for transgenic constructs. (DOC)

S5 Table Primers used for detecting the RNA level of *MKK7*. (DOC)

## Abbreviations

A: Ala
*bud1*: *bushy* and *dwarf1*
cMKK7: constitutively activated MKK7
Col: Arabidopsis Columbia
D: Asp
D6PKs: D6 PROTEIN KINASES
GFP: green fluorescent protein
GST: Glutathione S-transferase
^3^H-IAA: ^3^H- indole-3-acetic acid
HIS: histidine
HL: hydrophilic loop
LC-MS/MS: Liquid Chromatograph-Mass Spectrometer/ Mass Spectrometer
MAPK/MPK: mitogen-activated protein kinase
MAPKKK: MAPK kinase kinase
MBP: myelin basic protein
MKK: MAPK kinase
MKK7: MAP Kinase Kinase 7
PAT: polar auxin transport
PID: PINOID
PIN1: PIN-FORMED 1
PP2A: protein phosphatase 2A
PR: pathogenesis-related
Psm: *Pseudomonas syringae pv*. *Maculicola*
S337: Ser 337
SA: salicylic acid
S/T: Ser/Thr
