# Supplementary figures and images for: Mitogen-Activated Protein Kinase Cascade MKK7-MPK6 Plays Important Roles in Plant Development and Regulates Shoot Branching by Phosphorylating PIN1 in Arabidopsis
Source: PLoS Biol. 2016 Sep 12;14(9):e1002550. doi: 10.1371/journal.pbio.1002550 (PMC5019414; doi:10.1371/journal.pbio.1002550)

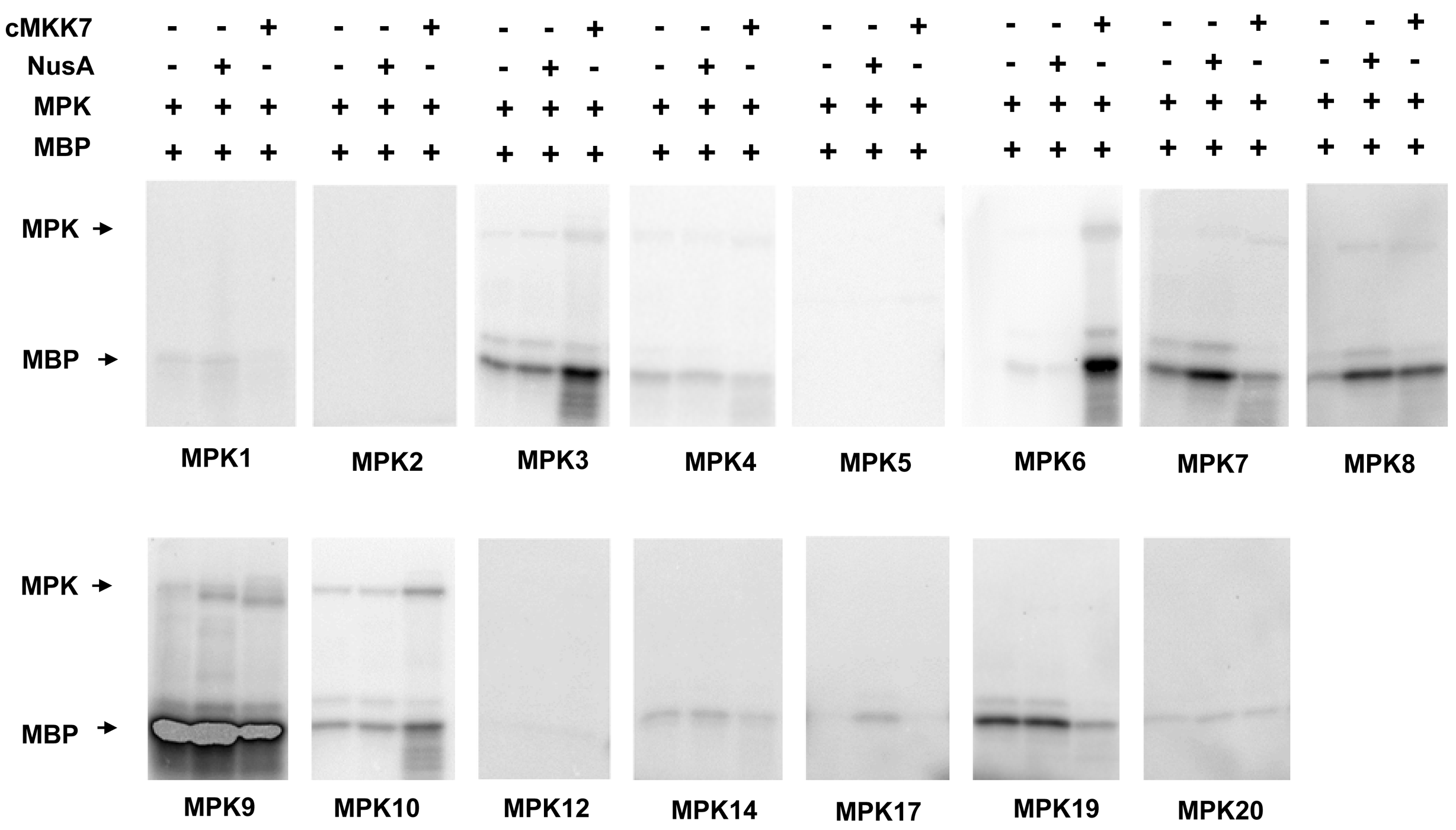

Supplement: S1 Fig — In vitro kinase assays for screening candidate substrates of MKK7 by constitutively activated MKK7 (cMKK7). The cMKK7 was incubated with various MPKs in the kinase reaction buffer, respectively. Aliquots of the samples were separated by SDS-PAGE and subjected to autoradiography. (TIF) [file pbio.1002550.s002.tif]

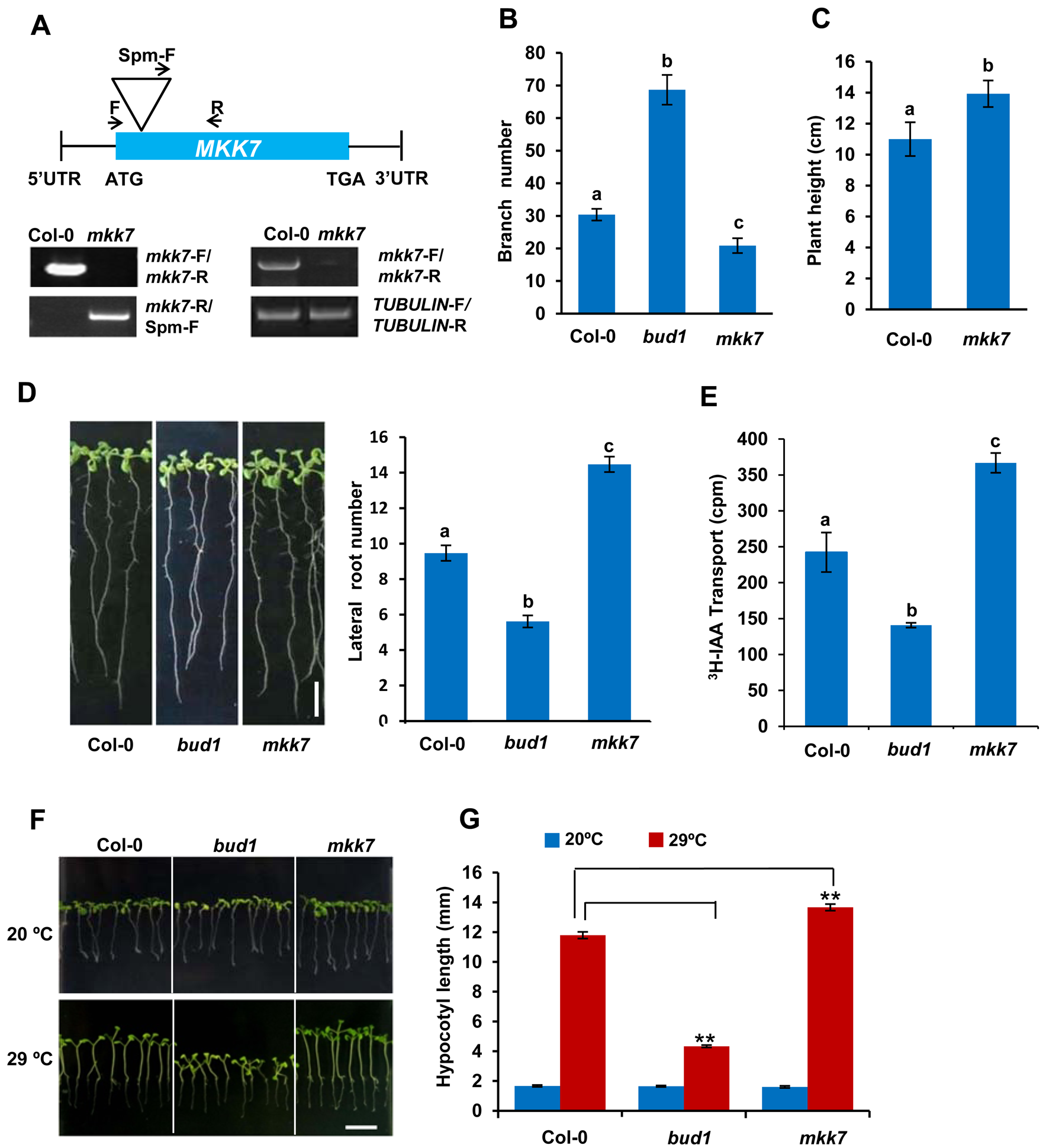

Supplement: S2 Fig — (A) Identification of the mkk7 mutant. Upper panel shows the diagram of MKK7 and the transposon insertion site. Lower panel showing PCR and RT–PCR analysis confirmed the absence of MKK7 in homozygous mkk7. (B) Branching phenotypes of 40-d-old Col-0, bud1, and mkk7 plants grown under the long day condition. Total branches were counted at 40 d. Data are shown as mean ± SE (n = 15). The difference significance was determined with Turkey’s HSD test (p < 0.01). (C) Plant height of 30-d-old Col-0, bud1, and mkk7 plants grown under the long day condition. Data are shown as mean ± SE (n = 16). The difference significance was determined with Turkey’s HSD test (p < 0.05). (D) Root architecture of Col-0, bud1, and mkk7 seedlings. Plants were grown vertically on 0.5 × MS containing 1% sucrose and 0.6% phytagel plates and photographed at 11 d after germination (left panel). Bars, 1 cm. Statistical analysis of lateral root number (right panel). Data are shown as mean ± SE (n = 38). The difference significance was determined with Turkey’s HSD test (p < 0.01). (E) Polar auxin transport assays of inflorescence stems. Values are means ± SE of six independent assays. The difference significance was determined with Turkey’s HSD test (p < 0.01). (F) Induction of hypocotyl elongation by high temperature. Wild-type and mutant seedlings were grown on 0.5 × MS solid media at 20°C and 29°C, respectively, and photographed at 9 d after germination. Bars, 10 mm. (G) Statistical analysis of high temperature-induced hypocotyl elongation. Values are means ± SE (n ≥ 22). ** differences for the mutants compared with wild-type are highly significant (p < 0.01). (TIF) [file pbio.1002550.s003.tif]

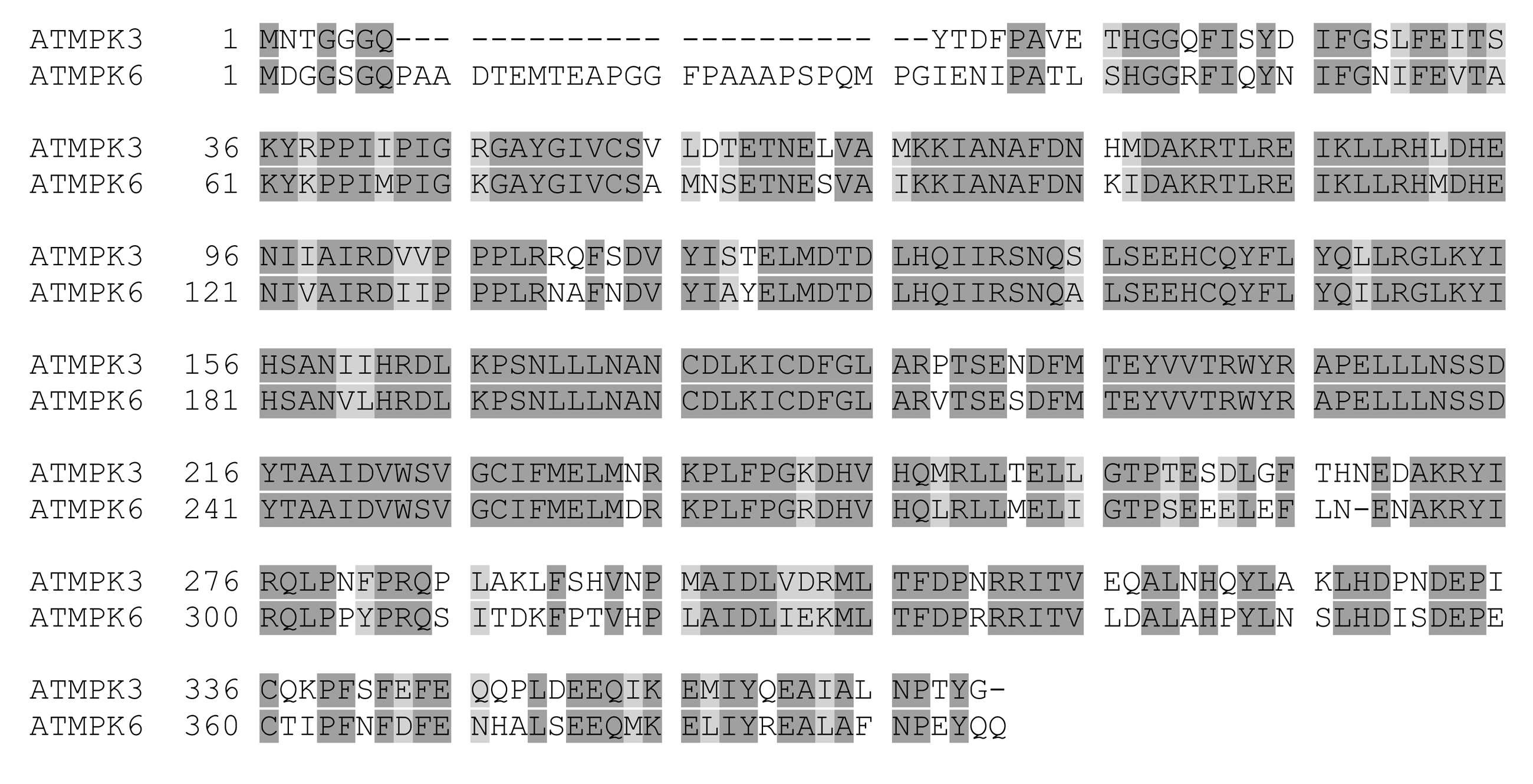

Supplement: S3 Fig — Analysis was performed by ClustalW2 online and edited by BioEdit software. (TIF) [file pbio.1002550.s004.tif]

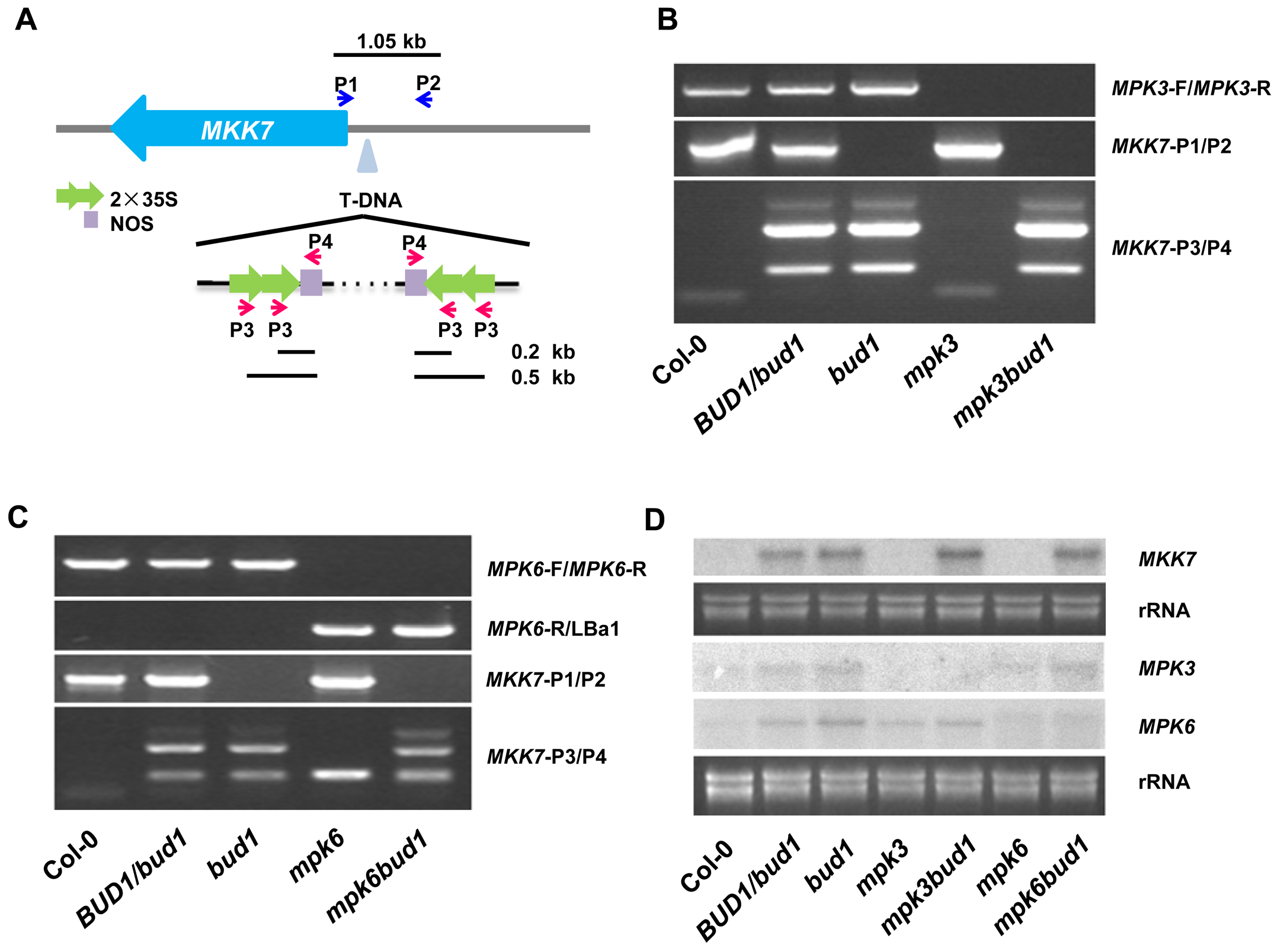

Supplement: S4 Fig — (A) Diagram showing binding sites of bud1genotyping primer. (B) Genotyping of the mpk3bud1 double mutant. (C) Genotyping of the mpk6bud1 double mutant. (D) Northern blot analysis showing the expression levels of MKK7, MPK3, and MPK6 in Col-0, BUD1/bud1, bud1, mpk3, mpk3bud1, mpk6, and mpk6bud1 plants. (TIF) [file pbio.1002550.s005.tif]

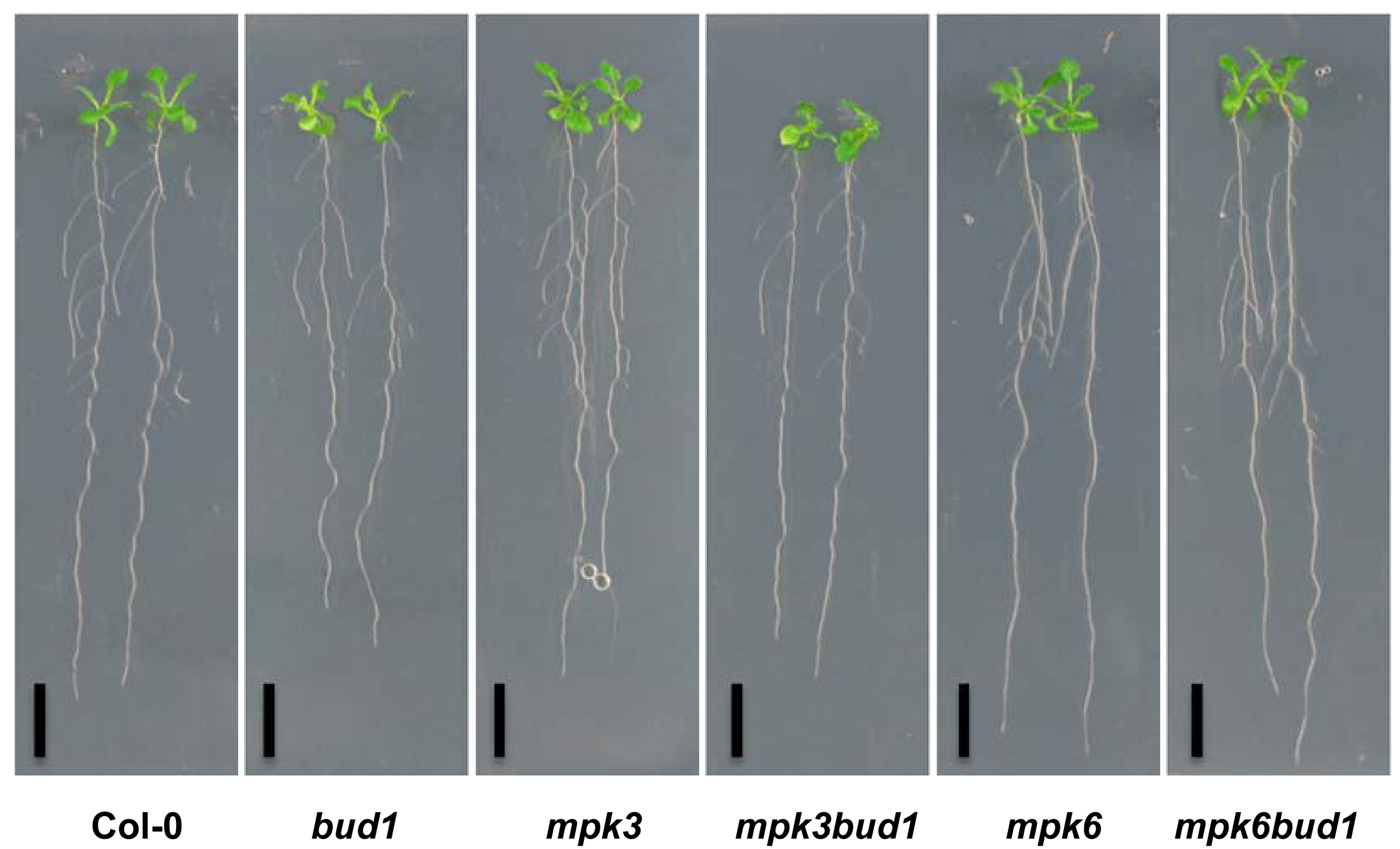

Supplement: S5 Fig — Plants were grown vertically on 0.5 × MS containing 1% sucrose and 0.6% phytagel plates and photographed at 12 d after germination. Bars, 1 cm. (TIF) [file pbio.1002550.s006.tif]

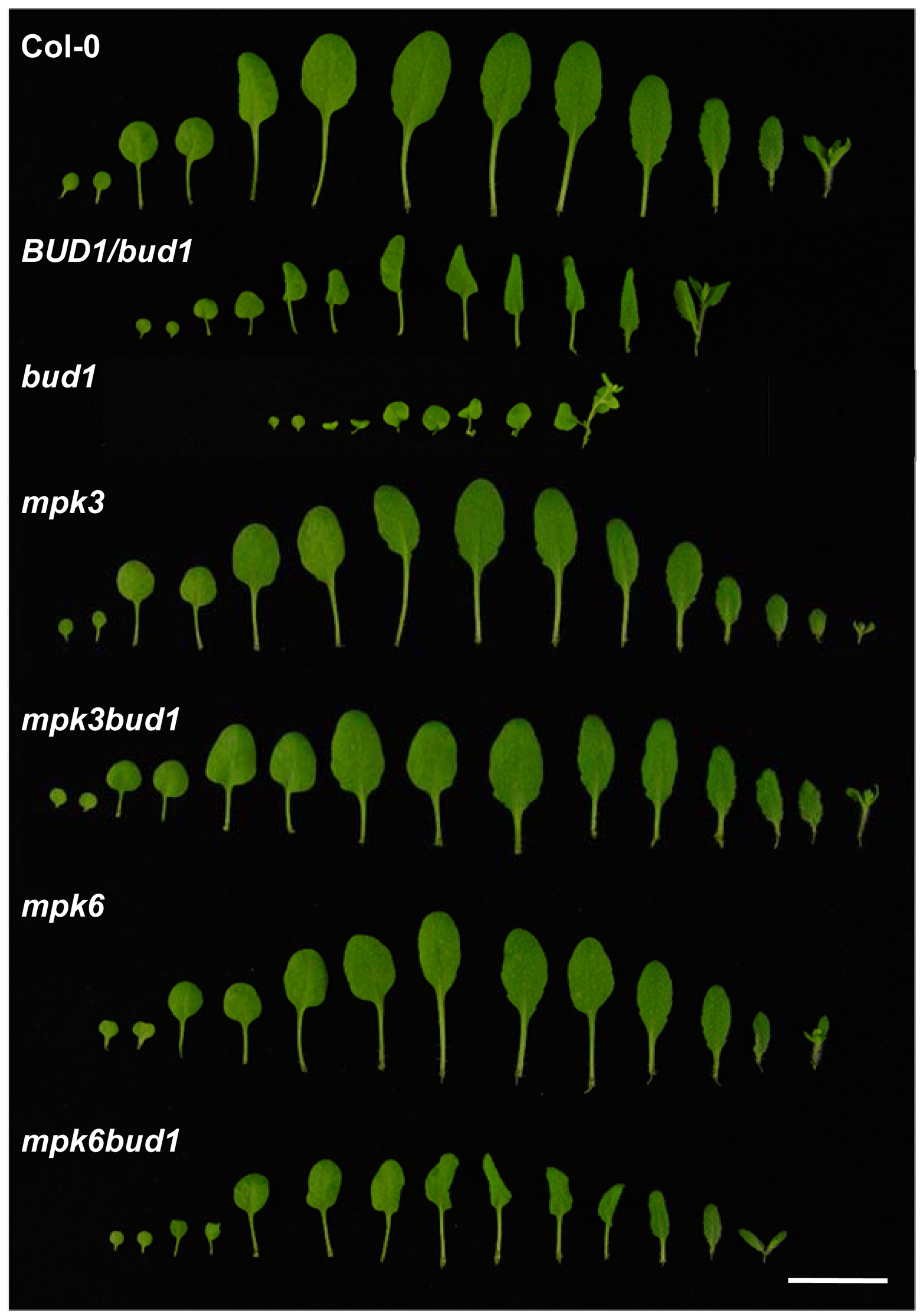

Supplement: S6 Fig — Bar, 2 cm. (TIF) [file pbio.1002550.s007.tif]

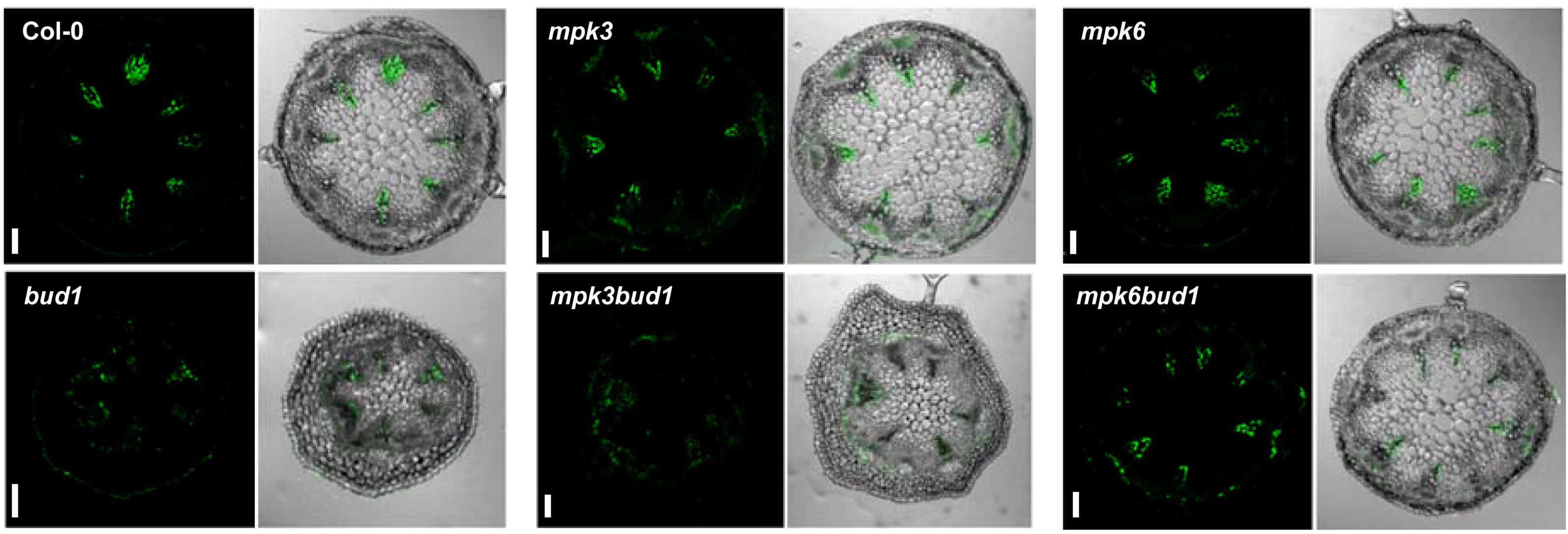

Supplement: S7 Fig — DR5-GFP activity in basal stem segments of Col-0, bud1, mpk3, mpk3bud1, mpk6, and mpk6bud1. Bars, 100 μm. (TIF) [file pbio.1002550.s008.tif]

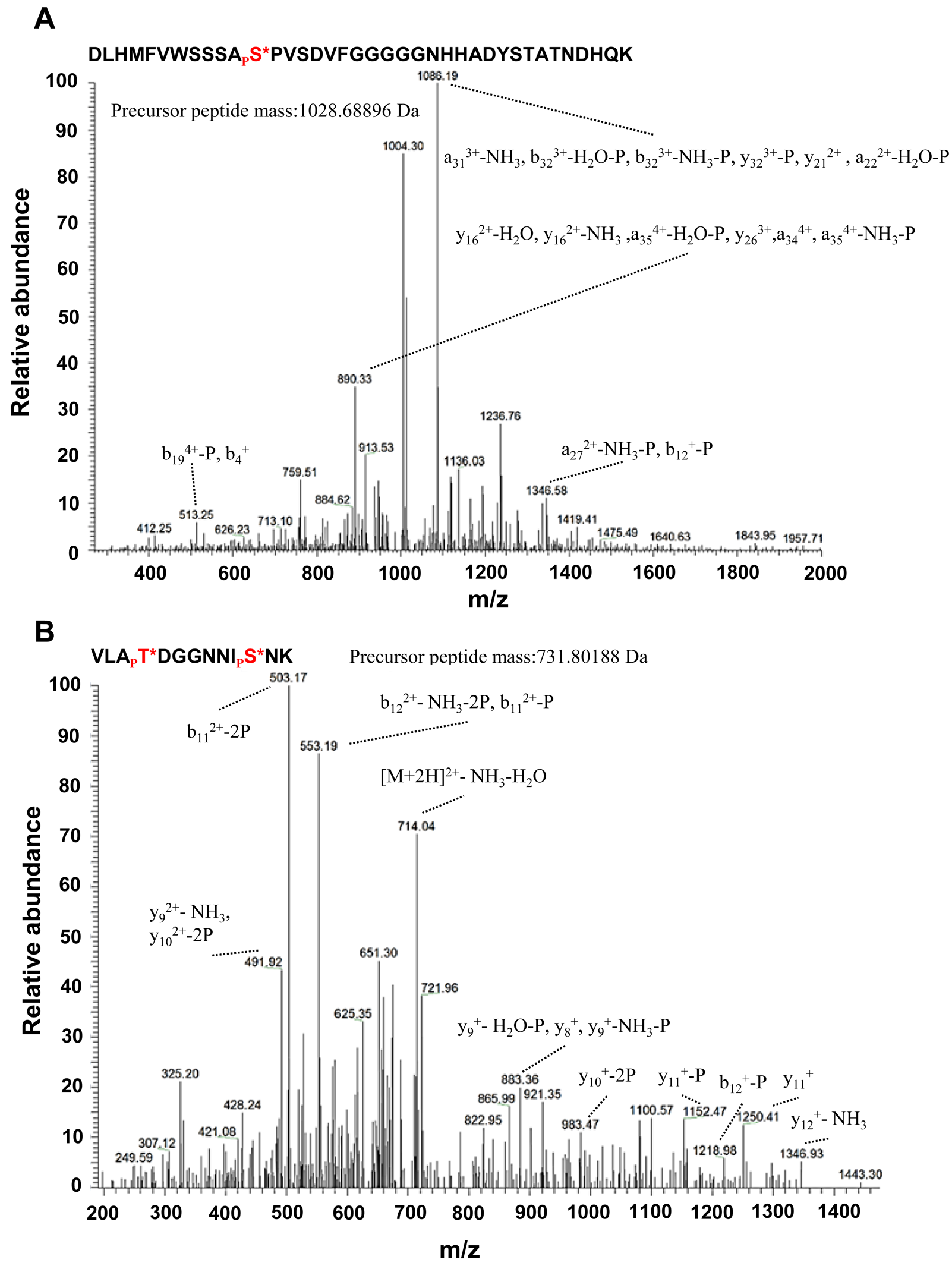

Supplement: S8 Fig — (A) Spectra for representative identified phosphopeptide S317. Asterisk represents the phosphate moiety. (B) Spectra for representative identified phosphopeptide T439 and S446. Asterisk represents the phosphate moiety. (TIF) [file pbio.1002550.s009.tif]

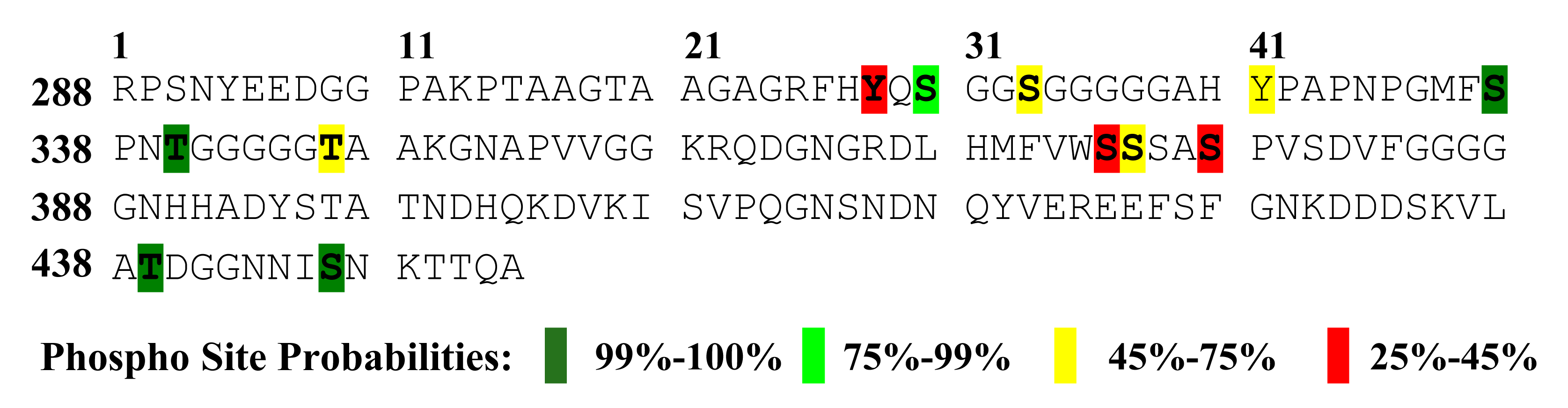

Supplement: S9 Fig — The phosphopeptides were analyzed using LTQ-Orbitrap elite with enabled multistage activation, and the phosphosites were assigned by the software protein discovery. The amino acid sequence of PINIHL mapped with the identified phosphosites and the confidence of each phosphosite (probability) was shown, respectively, with the indicated color. (TIF) [file pbio.1002550.s010.tif]

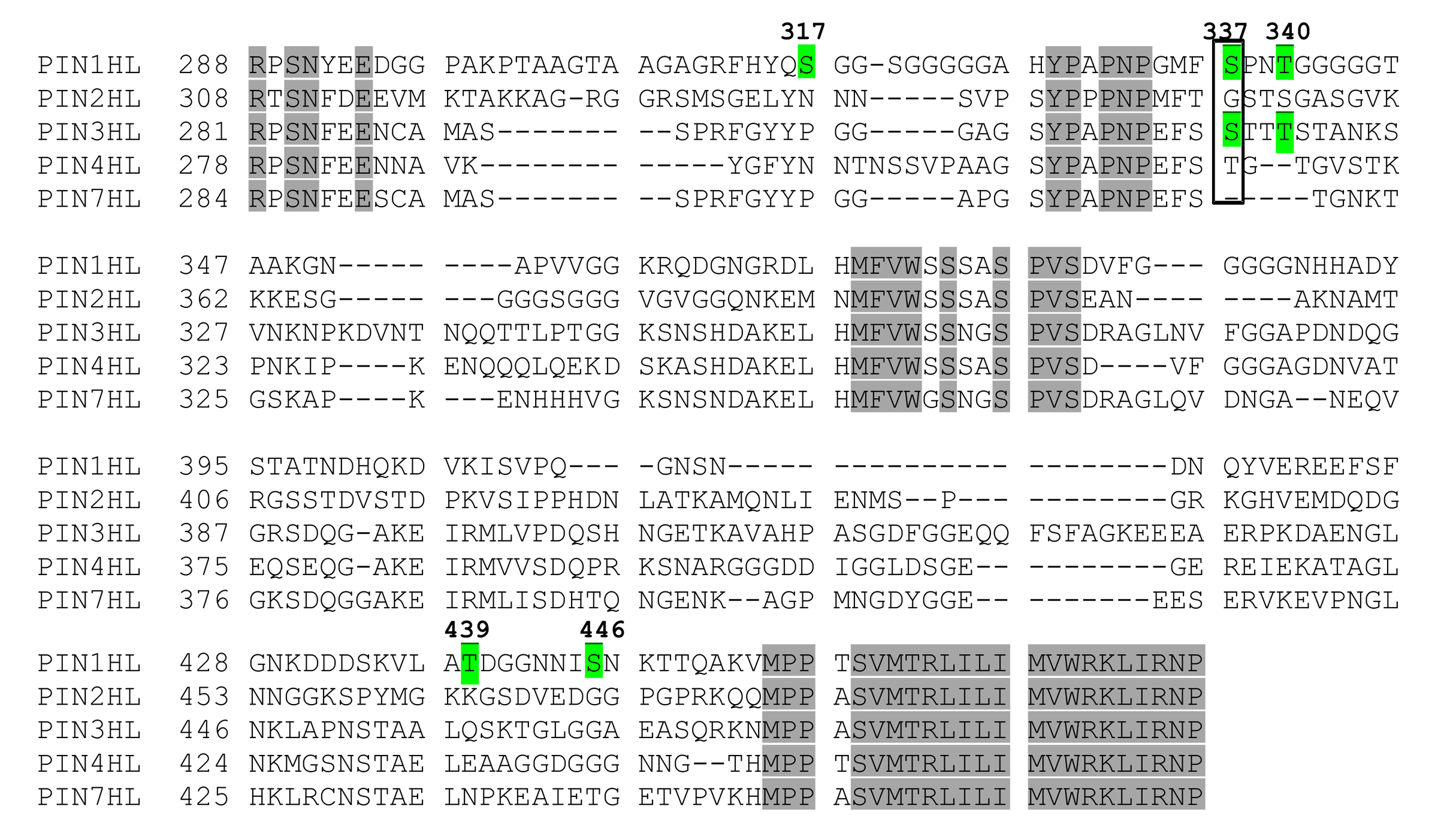

Supplement: S10 Fig — The main phosphorylation sites of PIN1HL are marked by bright green. Residues that are conserved in all five PINHLs are indicated with dark gray. (TIF) [file pbio.1002550.s011.tif]

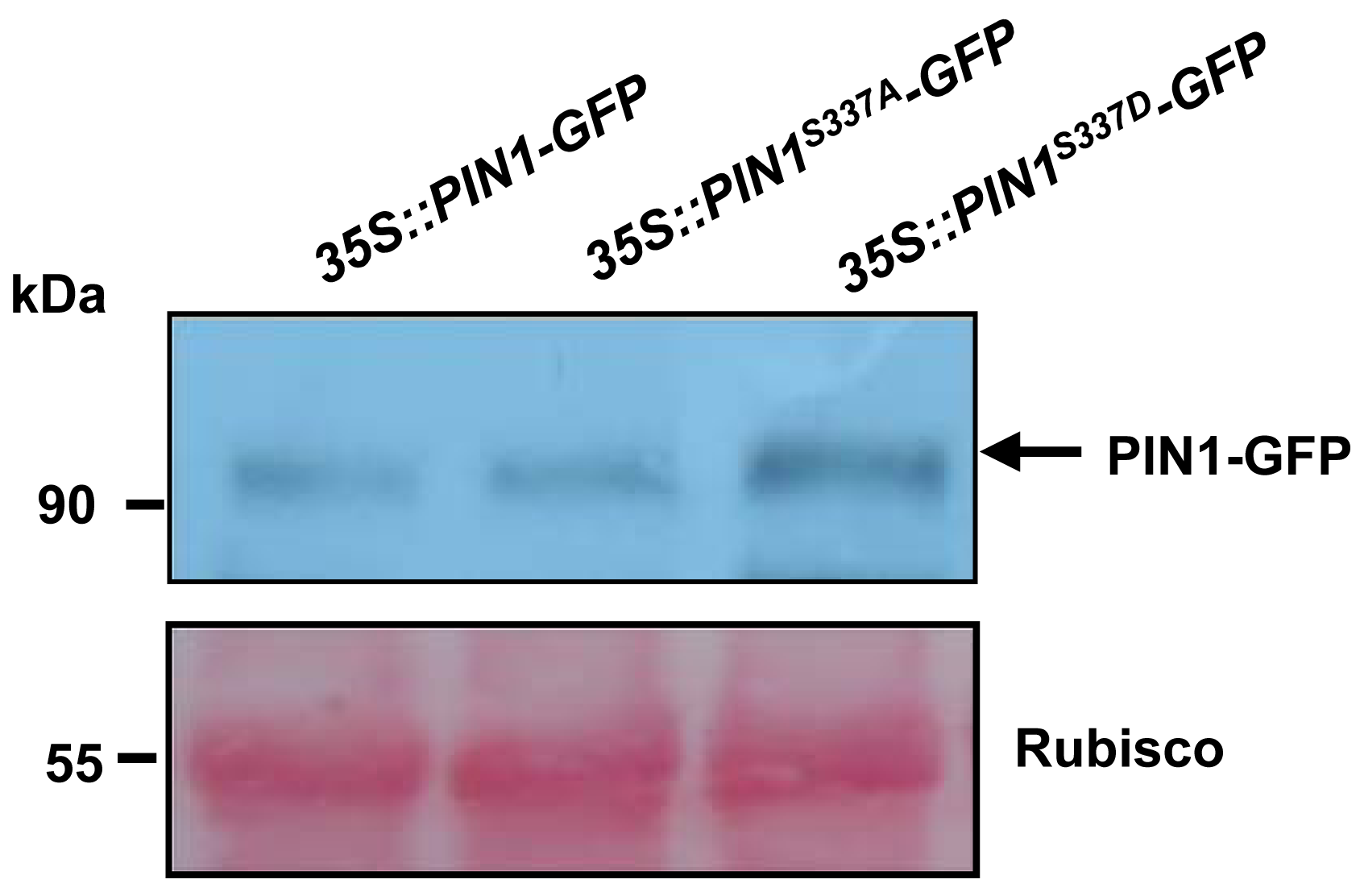

Supplement: S11 Fig — Total proteins were isolated from the same lines mentioned in Fig 7, and the PIN1-GFP protein levels were detected by immunoblotting with an anti-GFP monoclonal antibody. Rubisco staining was included for assessing equal protein loading. (TIF) [file pbio.1002550.s012.tif]

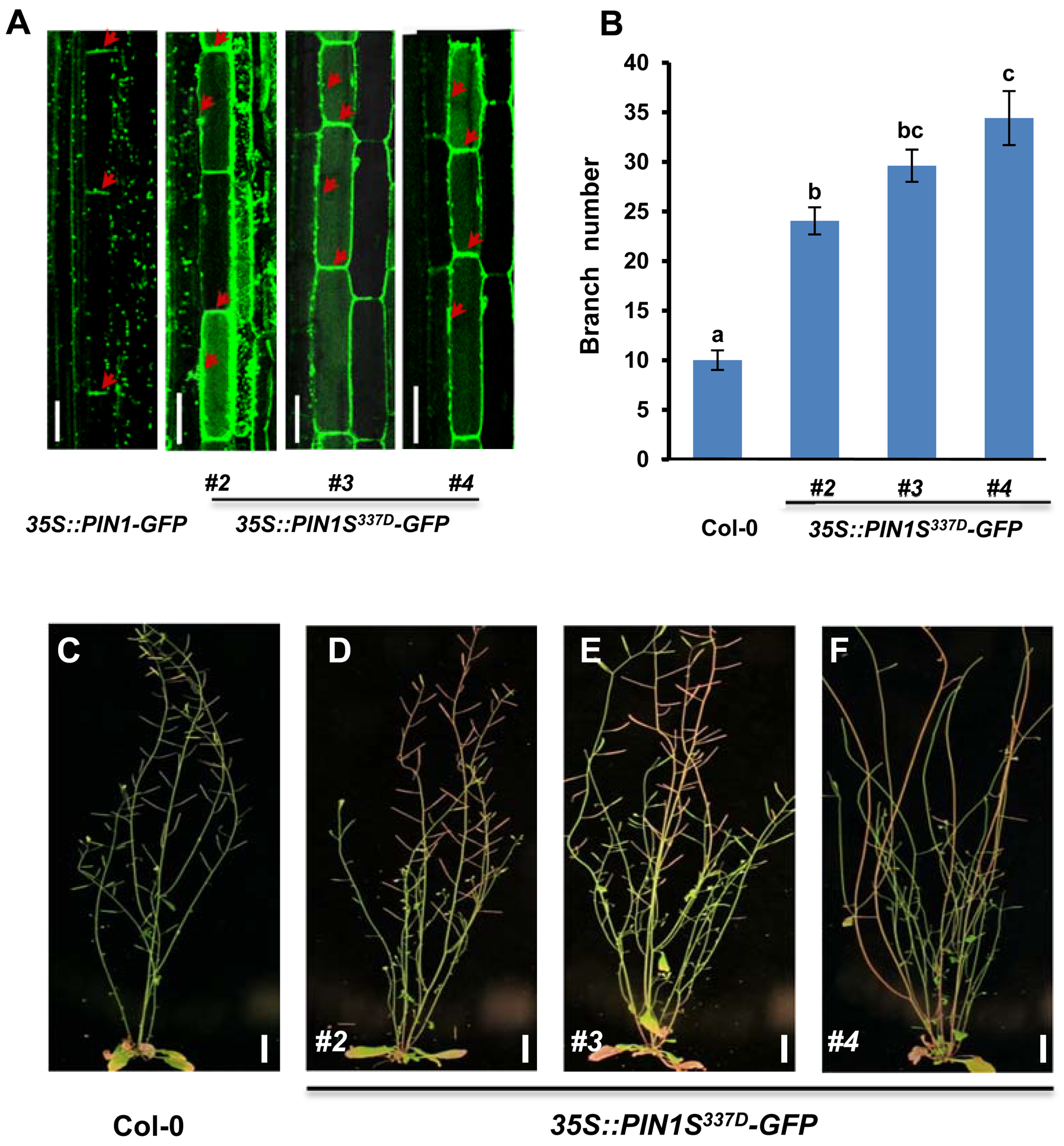

Supplement: S12 Fig — (A) PIN1-GFP localization in inflorescence stems of 35S::PIN1WT and three independent 35S::PIN1S337D-GFP transgenic lines. Red arrows indicate PIN1-GFP localization. Bars, 50 μm. (B) Comparison of branch number in wild-type plants and three independent 35S::PIN1S337D-GFP lines. Primary rosette-leaf branch (RI), secondary rosette-leaf branch (RII), primary cauline-leaf branch (CI), and secondary cauline-leaf branch (CII) were counted at 60 d. Data are shown as mean ± SE (n ≥ 17). The difference significance was determined with Turkey’s HSD test (p < 0.05). (C–F) Three transgenic lines harboring the phospho-mimicking PIN1S337D display branching phenotype. Bars, 5cm. (TIF) [file pbio.1002550.s013.tif]

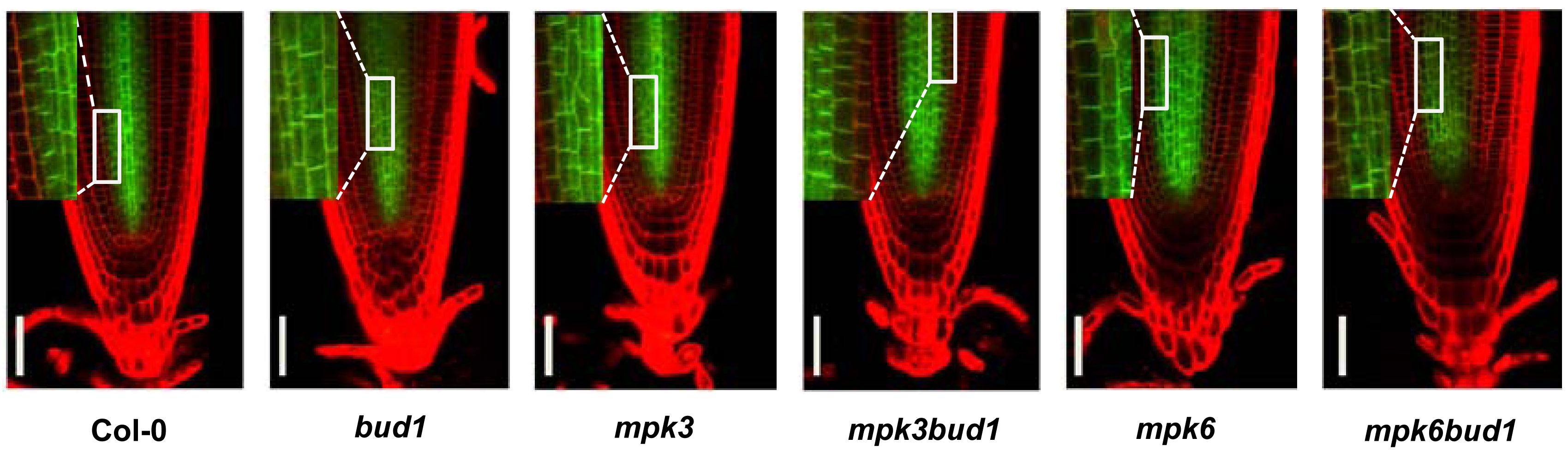

Supplement: S13 Fig — Localization of PIN1-GFP in longitudinal sections of 7-d-old roots of Col-0, bud1, mpk3, mpk3bud1, mpk6, and mpk6bud1. Bars, 50 μm. (TIF) [file pbio.1002550.s014.tif]

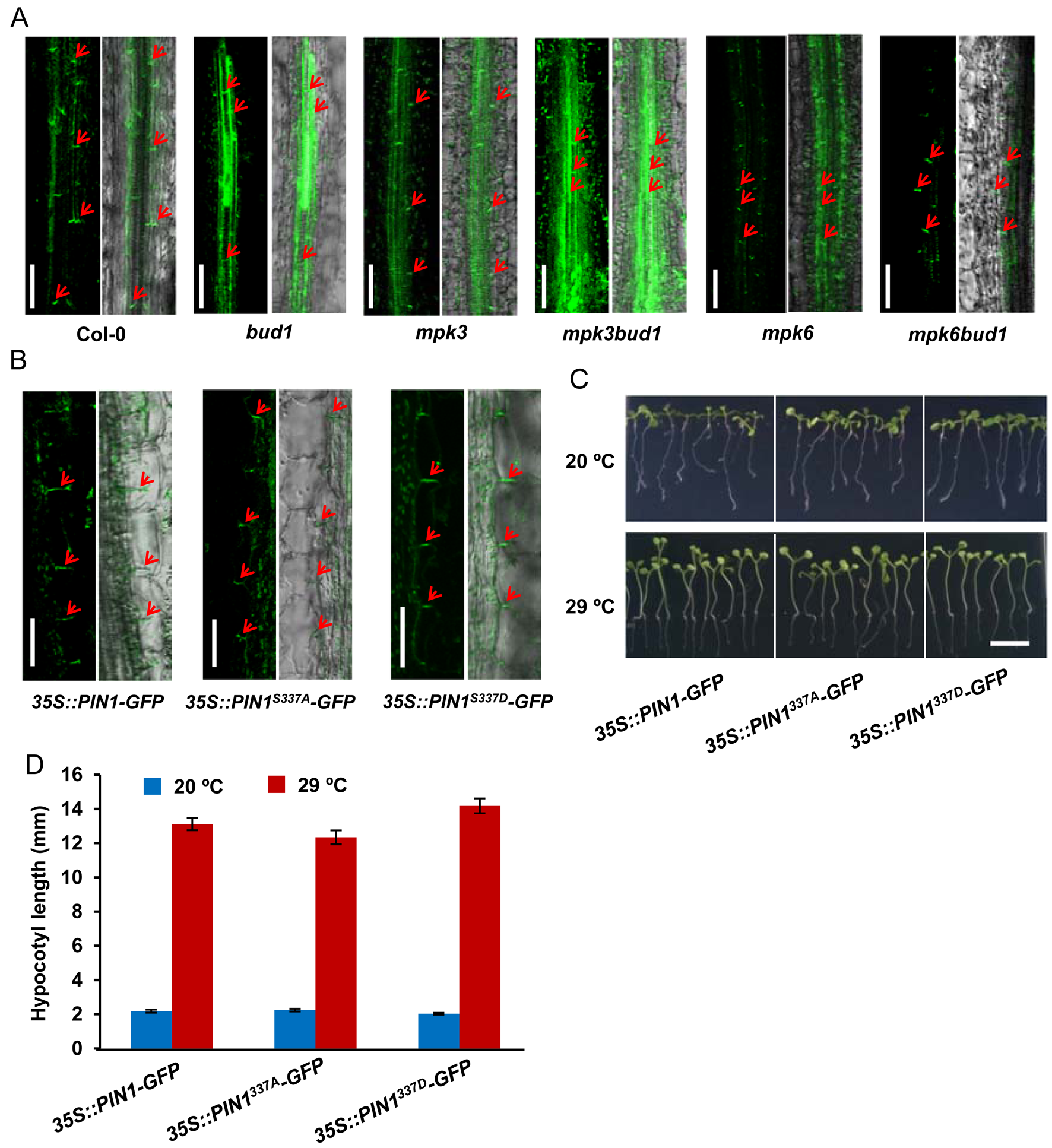

Supplement: S14 Fig — (A) Localization of PIN1-GFP in 7-d-old hypocotyls of Col-0, bud1, mpk3, mpk3bud1, mpk6, and mpk6bud1. Red arrows indicate PIN1-GFP localization. Bars, 50 μm. (B) PIN1-GFP localization in 7-d-old hypocotyls of 35S::PIN1WT-GFP, 35S::PIN1S337A-GFP, and 35S::PIN1S337D-GFP transgenic plants. Red arrows indicate PIN1-GFP localization. Bars, 50 μm. (C) Induction of hypocotyl elongation by high temperature. Wild-type and mutant seedlings were grown on 0.5 × MS solid media at 20°C and 29°C, respectively, and photographed at 9 d after germination. Bars, 10 mm. (D) Statistical analysis of high temperature-induced hypocotyl elongation. Values are means ± SE (n ≥ 15). (TIF) [file pbio.1002550.s015.tif]

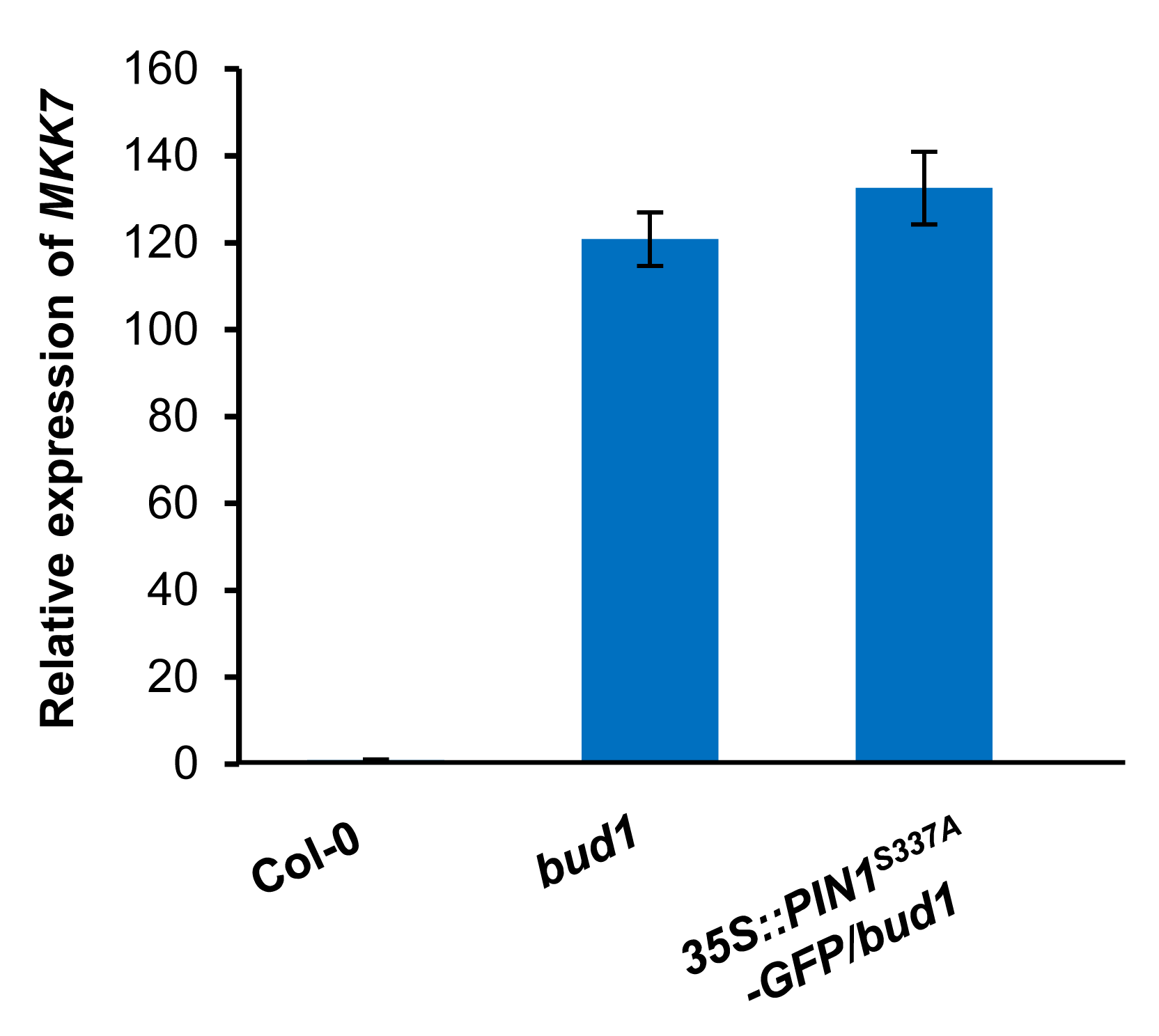

Supplement: S15 Fig — Relative expression of MKK7 in Col-0, bud1, and 35S::PIN1-GFPS337A/bud1 plants. Actin2 and Ubiquitin5 were used as an internal control and gene expression was normalized to the wild-type expression level. Data represent the average of three independent experiments ± SE. (TIF) [file pbio.1002550.s016.tif]

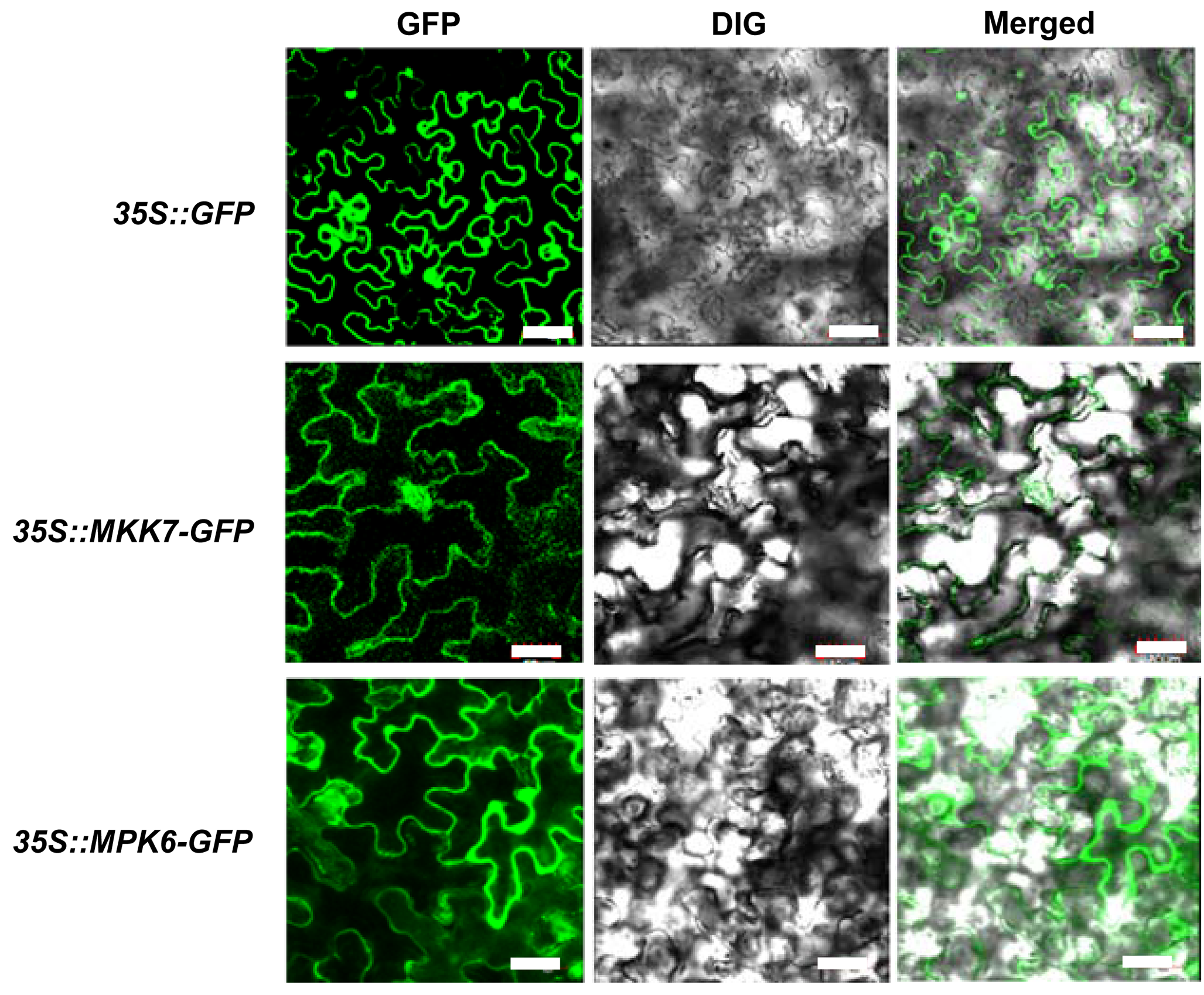

Supplement: S16 Fig — Representative confocal images of MPK6-GFP and MKK7-GFP localizations in tobacco epidermal cells. Bars, 50 μm. (TIF) [file pbio.1002550.s017.tif]
