# Supplementary material for: Mitogen-Activated Protein Kinase Cascade MKK7-MPK6 Plays Important Roles in Plant Development and Regulates Shoot Branching by Phosphorylating PIN1 in Arabidopsis
Source: PLoS Biol. 2016 Sep 12;14(9):e1002550. doi: 10.1371/journal.pbio.1002550 (PMC5019414; doi:10.1371/journal.pbio.1002550)
Supplement: S1 Table — (DOC) [file pbio.1002550.s018.doc]

**S1 Table. Primers used for genotyping homozygous mutants.**

| **Name** | **Sequence (5'-3')** |
| --- | --- |
| *MKK7*-P1 | TCTAGAAGCCGTGGAAATAGAAGAGAG |
| *MKK7*-P2 | CTGCAGCCGTAGGGTCAAGTGTGACTG |
| *MKK7*-P3 | ACCACGTCTTCAAAGCAAGTG |
| *MKK7*-P4 | TATGATAATCATCGCAAGACCG |
| LBa1 | TGGTTCACGTAGTGGGCCATCG |
| *MPK6-*F | TCATCTTCATCTCCCAAATGC |
| *MPK6-*R | AACGGGGACTAATTGAACTCC |
| *MPK3*-F | ATTTTTGTCAACAATGGCCTG |
| *MPK3-*R | TCTGCCTTTTCACGGAATATG |
| *mkk7-*F | CGATTCTGATAGGTAACACAAAGC |
| *mkk7-*R | CCACCGTCCATATACTCCATG |
| Spm-F | TACGAATAAGAGCGTCCATTTTAGAGTGA |
| *Tubulin*-F | TTTGGAGCCTGGGACTATGGAT |
| *Tubulin*-R | ACGGGGGAATGGGATGAGAT |
