# Supplementary material for: Mitogen-Activated Protein Kinase Cascade MKK7-MPK6 Plays Important Roles in Plant Development and Regulates Shoot Branching by Phosphorylating PIN1 in Arabidopsis
Source: PLoS Biol. 2016 Sep 12;14(9):e1002550. doi: 10.1371/journal.pbio.1002550 (PMC5019414; doi:10.1371/journal.pbio.1002550)
Supplement: S2 Table — (DOC) [file pbio.1002550.s019.doc]

**S2 Table. Primers used for construction of MPK recombinant proteins.**

| **Name** | **Sequence (5'-3')** |
| --- | --- |
| *MPK1-*F | gaattcATGGCGACTTTGGTTGATCCT |
| *MPK1-*R | ctcgagTACCCGTACAAACAAGACTTGAGC |
| *MPK2*-F | GAATTCATGGCGACTCCTGTTGATC |
| *MPK2-*R | GTCGACAAACTCAGAGACCTCATTGTTG |
| *MPK3-*F | ggatccATGAACACCGGCGGTGGCCA |
| *MPK3-*R | gaattcTTAACCGTATGTTGGATTGA |
| *MPK4-*F | gaattcATGTCGGCGGAGAGTTGTTT |
| *MPK4-*R | gtcgacTTACACTGAGTCTTGAGGAT |
| *MPK5-*F | gtcgacagATGGCGAAGGAAATTG |
| *MPK5-*R | gcggccgcTCCCCTTCTTAAATAATG |
| *MPK6-*F | ggatccATGGACGGTGGTTCAGG |
| *MPK6-*R | gtcgacATCTCATCTTCATCTCCC |
| *MPK7-*F | gaattcATGGCGATGTTAGTTGAGC |
| *MPK7-*R | gtcgacTTAGGCATTTGAGATTTCA |
| *MPK8-*F | GAGCTCATGGGTGGTGGTGGGAATCT |
| *MPK8-*R | GCGGCCGCTTAAGAATTGTGAAGAGAAG |
| *MPK9-*F | gaattcATGGATCCTCATAAAAAGGT |
| *MPK9*-R | gtcgacTAAAGTGTGGAGAGCCGCGA |
| *MPK10-*F | gaattcATGGAGCCAACTAACGATGCT |
| *MPK10-*R | ctcgagTCAATCATTGCTGGTTTCAGG |
| *MPK12-*F | gaattcATGTCTGGAGAATCAAGCTCTG |
| *MPK12-*R | ctcgagTCAGTGGTCAGGATTGAATTTG |
| *MPK14-*F | gaattcATGGCGATGCTAGTTGATCCTC |
| *MPK14-*R | ctcgagTTAAGCTCGGGGGAGGTAATG |
| *MPK17-*F | cccgggtATGTTGGAGAAAGAGTTTTT |
| *MPK17-*R | gcggccgcTTATGACACTGCAGAGGAGA |
| *MPK19-*F | ggatccATGGAGTTTTTCACTGAG |
| *MPK19-*R | gaattctTAAGACATGCCATACCC |
| *MPK20-*F | ggatccATGCAGCAAGATAATCGCAA |
| *MPK20-*R | gtcgacTTAGTACATCTTTGACATAC |
