# Supplementary material for: Mitogen-Activated Protein Kinase Cascade MKK7-MPK6 Plays Important Roles in Plant Development and Regulates Shoot Branching by Phosphorylating PIN1 in Arabidopsis
Source: PLoS Biol. 2016 Sep 12;14(9):e1002550. doi: 10.1371/journal.pbio.1002550 (PMC5019414; doi:10.1371/journal.pbio.1002550)
Supplement: S3 Table — (DOC) [file pbio.1002550.s020.doc]

**S3 Table. Primers used for construction of PIN1 site-directed mutagenesis.**

| **Name** | **Sequence (5'-3')** |
| --- | --- |
| S317A-F | AGCTGGGAGGTTTCATTATCAAGCTGGAGGAAGTG |
| S317A-R | CACTTCCTCCAGCTTGATAATGAAACCTCCCAGCT |
| S337A-F | GCCGAACCCAGGGATGTTTGCGCCCAACAC |
| S337A-R | GTGTTGGGCGCAAACATCCCTGGGTTCGGC |
| T340A-F | GTTTTCGCCCAACGCTGGCGGTGGTGG |
| T340A-R | CCACCACCGCCAGCGTTGGGCGAAAAC |
| T439A-F | GATGATAGCAAAGTATTGGCAGCGGACGGTGGG |
| T439A-R | CCCACCGTCCGCTGCCAATACTTTGCTATCATC |
| S446A-F | CGGACGGTGGGAACAACATAGCCAACAAAACGACGCAG |
| S446A-R | CTGCGTCGTTTTGTTGGCTATGTTGTTCCCACCGTCCG |
