# Supplementary material for: Mitogen-Activated Protein Kinase Cascade MKK7-MPK6 Plays Important Roles in Plant Development and Regulates Shoot Branching by Phosphorylating PIN1 in Arabidopsis
Source: PLoS Biol. 2016 Sep 12;14(9):e1002550. doi: 10.1371/journal.pbio.1002550 (PMC5019414; doi:10.1371/journal.pbio.1002550)
Supplement: S4 Table — (DOC) [file pbio.1002550.s021.doc]

**S4 Table. Primers used for transgenic constructs.**

| **Name** | **Sequence (5'-3')** |
| --- | --- |
| *PIN1-GFP*-F | GGGGACAAGTTTGTACAAAAAAGCAGGCTTAATGATTACGGCGGCGGAC |
| *PIN1-GFP*-R  *MPK6-GFP*-F  *MPK6-GFP*-R  *MKK7-GFP*-F  *MKK7-GFP*-R | GGGGACCACTTTGTACAAGAAAGCTGGGTATCATAGACCCAAGAGAAT  AAGTCGACATGGACGGTGGTTCAGGTC  AAGATATCGTTGCTGATATTCTGGATTGAA  CGCGGATCCatggctcttgttcgtaaac  CCCAAGCTTAAGACTTTCACGGAGAAAAG |
